# Competencies and Ethical Governance for Psychedelic‑Assisted Therapies: A Practice‑Embedded Quality‑Improvement Framework

**DOI:** 10.1111/jep.70556

**Published:** 2026-08-10

**Authors:** Shannon Dames, Keith Williams, Allan Campbell, Wes Taylor, Marti Harder, Michelle Gagnon, Geraldine Manson, Pamela Kryskow

**Affiliations:** ^1^ Psychedelic‐Assisted Therapy Graduate Certificate (PaTGC) Vancouver Island University (VIU) Nanaimo British Columbia Canada; ^2^ Roots to Thrive Society for Psychedelic Therapy Nanaimo British Columbia Canada; ^3^ Athabasca University Athabasca Alberta Canada; ^4^ First Nations Technical Institute Tyendinaga Mohawk Territory Ontario Canada; ^5^ Kyuquot/Cheklesahht First Nation Vancouver British Columbia Canada; ^6^ Faculty of Health Sciences and Human Services VIU Nanaimo British Columbia Canada; ^7^ School of Nursing University of British Columbia Vancouver British Columbia Canada

## Abstract

**Rationale:**

Psychedelic‑assisted therapy (PaT) is a rapidly expanding practice area that spans multiple disciplines and regulated and unregulated settings, without shared ownership by any single profession. As a result, training and service delivery are advancing more rapidly than the development and evaluation of research‑informed governance frameworks from public and academic bodies. Consequently, practitioners may rely on guidance that varies in transparency, evidence base and methodological rigour, leading to inconsistent safety and ethical expectations.

**Aims:**

In response to this gap, we describe a competency framework developed over a 4‑year, practice‑embedded quality improvement (QI) process focused on governance, safety and accountability.

**Methods:**

Within a post‐graduate PaT programme at a Canadian university, we used an iterative QI approach that integrated evidence‑informed literature with recurring feedback from learners, faculty, supervised practice partners and cultural knowledge‑keeper guidance. This process is centred on developmental learning, cultural responsiveness and safeguards appropriate to non‑ordinary states of consciousness.

**Results:**

The framework centres on both *doing* (technical and clinical aspects of practice) and *being* (relational presence and ethical stance). These dimensions are supported by a programme‑level Code of Ethics addressing safeguards related to safety, confidentiality and privacy, transparency, equity and cultural accountability, competence, environmental stewardship and power and boundary management, including touch and sexual boundaries. The framework operationalizes five competency domains: (1) therapeutic presence and trustworthiness; (2) culture and identity; (3) historical, political, and regulatory literacy; (4) evidence‑informed clinical knowledge; and (5) a trauma‑ and equity‑informed therapeutic process spanning screening, preparation, informed consent, team roles and integration.

**Conclusion:**

Rather than a fixed consensus standard or externally validated competency model, this framework offers a practice‐embedded, QI‐derived design logic that can be locally adapted across diverse contexts. Its contribution is governance‐oriented: clarifying ethical guardrails, developmental competencies and revision processes for future implementation and evaluation.

## 1 Introduction

After decades of prohibition, psychedelics are re‑emerging in Western health and research contexts, with growing activity across both regulated and unregulated settings [1, 2]. Interest, investment and clinical uptake are outpacing publicly vetted practice standards and safeguards. While many private, community‑based and lineage‑informed settings hold established ethical norms, publicly accessible, research‑informed standards remain in development across training and practice contexts. In some areas, regulatory bodies have begun to clarify expectations for clinical research with psychedelic drugs [3], yet consistent standards emerging from public bodies and real‑world practice and training environments remain limited [4].

This gap is compounded by the multidisciplinary nature of psychedelic‑assisted therapy (PaT). PaT does not sit fully within any single professional domain; rather, it draws on—and is variably shaped by—clinical psychology, psychiatry, psychotherapy, somatic and relational therapies, neuroscience, ethics, education, spiritual care and community‑based and cultural knowledge systems. As a result, no one field fully *owns* PaT training, practice or governance. While this plurality is a strength, it also complicates the development, evaluation and governance of shared competency expectations, accountability and public‑facing standards, particularly in training contexts that sit outside traditional licensure or regulatory frameworks [4].

Further, PaT involves non‑ordinary states of consciousness that can heighten suggestibility, relational vulnerability and boundary risk, placing increased ethical and relational demands on practitioners and training programmes [5]. In this context, training and competency frameworks function not only as educational benchmarks but also as public‑facing safeguards that support clinical integrity, accountability and harm prevention as the field continues to scale [6].

In response to this gap within our training context, we describe a practice‑embedded quality improvement (QI) effort conducted in a Canadian public educational setting, resulting in:

a. A competency framework (Appendix S1),
b. Associated competency standards (Appendix S2) and
c. A Code of ethics (Appendix S3).

To maintain analytic focus, we examine how the framework was developed and refined through practice‑embedded QI, rather than providing a full account of the pedagogical or cultural frameworks that inform the broader PaT programme.

### 1.1 Bridging to Traditional Teachings and Cultural Context

Our attention to ethics and cultural humility centres on accountability to the historical contexts in which psychedelic medicines have long been held and used as healing practices, as well as to the ongoing impacts of colonial extractivism shaping what is often referred to as the ‘psychedelic renaissance’ [7, 8]. Within this orientation, the programme integrates guidance from the resident Elder, including the teaching ‘Naut sa Mawt—working together, as one mind and one spirit’ (Elder Manson, personal communication, 2022), alongside frameworks for respectful knowledge engagement such as Two‑Eyed Seeing (*Etuaptmumk*), as articulated by Mi'kmaw Elder Albert Marshall and described by Bartlett et al. [9] and Third Space Theory [10]. Clear agreements regarding how knowledge is shared, situated and applied distinguish cultural engagement from appropriation and align with broader calls for reciprocity, consent and cultural accountability in psychedelic research and practice. These references do not claim to represent Indigenous knowledge systems in full, nor to suggest authorization beyond the specific relational contexts in which guidance was offered.

### 1.2 Adapting and Evolving the Framework

This framework is intended for educators, supervisors and programme designers engaged in PaT training and governance. Core ethical and safety commitments are fixed, while content and application are revised only when recurring themes arise across routine educational contexts and supervised practice sites. This revision logic reflects the framework's broader stance: ethics and governance are treated as integral to competence, not secondary to technical skill. These commitments are refined through emerging evidence and practice‐informed education, rather than treated as static guidance.

## 2 Methods

### 2.1 Design, Epistemological Orientation and Scope

We report on a practice‑embedded QI initiative that developed competency standards and a Code of Ethics for PaT. The approach was iterative and grounded in qualitative, interpretivist enquiry and social constructionism, viewing knowledge as co‑created and situated within cultural and historical contexts [11]. Consistent with QI and programme‑evaluation traditions, rather than testing clinical efficacy or making causal claims, we document the design logic, feedback processes and decision rules used to refine the framework over time, drawing on established QI reporting guidance (SQUIRE 2.0) to support clarity and transparency in describing these processes [12].

### 2.2 Programme Development Context

Since 2020, faculty have advanced PaT programming through an integrated system in which service delivery, practice‑based research and education inform one another. In 2022, Vancouver Island University launched an accredited, year‑long postgraduate certificate as a QI initiative to iteratively develop a PaT competency framework for an interdisciplinary training context. Experienced practitioner‑learners contributed through application, feedback and refinement of the framework, informed by established PaT guidelines, relevant literature and explicit commitments to ethical practice, cultural accountability and relational safety, with guidance from the resident Elder and multidisciplinary representatives.

### 2.3 Setting and Context

**Figure 1:**
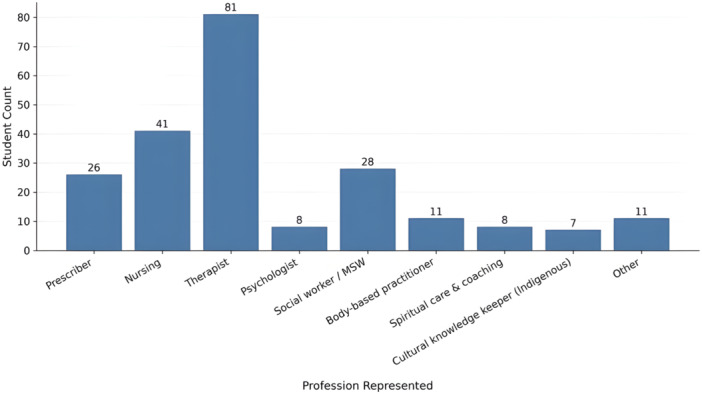
Student professional backgrounds across the 2022–2025 cohorts. Bars show the number of enroled students who reported each professional background in programme applications for the 2022–2025 cohorts. Individuals could select more than one background; counts reflect category representations and therefore exceed the unique headcount. Categories were collapsed from applicant‑provided role labels (e.g., ‘therapist/counsellor’ grouped as Therapist). Data represent enrolment at programme start; late withdrawals are not excluded.

The framework was developed and implemented within a postgraduate PaT programme at Vancouver Island University (launched 2022; year‑long certificate). Across four cohorts, learners used the standards for self‑assessment, peer feedback and practice‑based learning, including supervised practicum environments. Cohort composition is summarized in Figure 1, and enrolment/retention trends appear in Table 1 (attrition < 10% annually), supporting iterative refinement within a stable practice setting.

**Table 1:** Enrolment data for 219 students between 2022 and 2025.

Academic year	Approx. cohort size	Attrition rate
Year 1	61 students	< 10%
Year 2	55 students	< 10%
Year 3	63 students	< 10%
Year 4	40 students	< 10%

*Note:* Historical enrolment and retention data from the programme's 4 years of delivery.

### 2.4 Stakeholders, Inputs and Implementation Feedback Sources

**Figure 2:**
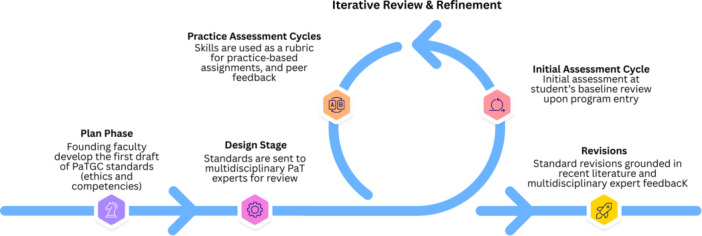
Iterative PaT standard development process. Revisions were informed by recurring feedback patterns and implemented at thematic saturation.

Standards were co‐created by faculty, multidisciplinary learners and supervised practice partners, with expert and cultural knowledge‐keeper consultation. Feedback was gathered through competency‐embedded assignments and rubrics, baseline and end‐of‐course self‐assessments, peer and faculty feedback, supervised practice observations, learner reflections and periodic alumni practice‐relevance review. Feedback was reviewed in aggregate to identify recurring issues related to clarity, usability, safety, cultural responsiveness, role boundaries and assessment feasibility. The revision decision flow is outlined in Figure 2.

### 2.5 Revision Decision Rules and QI Data Handling

Competencies were embedded in assignment rubrics and used in baseline and end‑of‑course self‑assessments. Learners and alumni provided in‑document feedback that was reviewed periodically. Faculty and programme leads reviewed feedback against three criteria: recurrence across cohorts or feedback sources, implications for learner safety or ethical accountability and alignment with the framework's developmental and governance purpose. Changes were made only when recurring patterns indicated a need to clarify competency language, learning outcomes, assessment expectations or ethical guardrails, ensuring that revisions were grounded in consistent feedback rather than isolated comments.

No individual‑level or clinical outcome data were analysed. All feedback was reviewed in aggregate as part of routine educational QI and programme development activities. Data use was limited to curriculum refinement and instructional decision‐making, consistent with quality‑assurance initiatives. As an educational QI initiative, the process did not include external validation, psychometric testing or evaluation of learner, client or clinical outcomes.

To operationalize the standards, we used three tools: a Skillfulness Competency Grid (Appendix S1), which supports formative self‑reflection and peer/faculty feedback; a student‑facing Skillfulness Assessment (Appendix S2), which organizes competencies and learning outcomes for instructional design and assessment alignment; and the Code of Ethics (Appendix S3), which provides the shared agreements and guardrails for reflection, feedback and decision‑making in training and supervised practice.

### 2.6 Ethical Considerations

This initiative constituted educational quality improvement rather than human‑subjects research. No individual‑level clinical or outcome data were collected, and all feedback was reviewed in aggregate for programme refinement purposes. In line with established guidance distinguishing quality improvement from research, formal research ethics board review was not required ([12]; SQUIRE 2.0).

## 3 Results

### 3.1 Competency Standards (Summarizing Appendix S1 & S2)

Competency‑based education (CBE) focuses on assessing clearly defined learning outcomes, which are typically developed in collaboration with professional or industry stakeholders, and supports learners in demonstrating mastery through flexible timelines and individualized learning pathways [13].

CBE models commonly feature flexible assessment and learner‑centred pacing and are adaptable across diverse educational contexts [14, 15]. However, CBE has been critiqued through Foucault's concept of disciplinary power, which suggests that competency frameworks can reinforce surveillance, normalization and standardization, potentially constraining creativity and reproducing harmful professional norms [16, 17].

Consistent with a practice‑embedded QI approach, the competency standards reflect knowledge as co‑created through social interaction and situated within cultural and historical contexts, an orientation aligned with constructivist evaluation traditions [11]. Extending beyond traditional CBE models, the framework integrates both *doing* (observable practices and actions) and *being* (relational presence expressed in conduct, attunement and responsiveness to others)—the latter understood as embodied, interactional capacities rather than private inner states [18].

In this context, *being* is treated as a professional and relational capacity expressed through observable conduct, reflective practice and responsiveness to feedback, rather than as an assessment of private inner states or personal character. Framed this way, the integration of *being* alongside technical *doing* supports training standards that function as more than technical checklists in practice settings where non‑ordinary states of consciousness can intensify vulnerability, relational dynamics and boundary risk. By emphasizing both embodied understanding and reflective engagement, the model directs attention to how practitioners notice, interpret and respond within real‑world relational situations, promoting reflexivity, ethical enquiry and relational integrity alongside technical competence—capacities that directly influence safety and therapeutic outcomes in PaT [16]. This orientation also aligns with transformative learning traditions that emphasize critical reflection on assumptions, meaning‑making and professional identity in the development of ethical practice [19].

### 3.2 Integral Theory and Depth–Breadth Development

Mastery here is different from perfection—it reflects sufficient skill, awareness and grounding to navigate the real‑world complexity of PaT while prioritizing safety, consent, boundaries and power dynamics. Across all competencies, skillfulness is assessed developmentally along these two complementary dimensions—depth (vertical; *being*) and breadth (horizontal; *doing)*—drawing on integrative developmental perspectives [20]. Importantly, depth development is approached formatively rather than as a measure of personal character or internal states; assessment focuses on observable relational capacities, reflective engagement and responsiveness to feedback within supervised and peer‑supported learning contexts. Table 2 summarizes the stage models used to support self‑assessment, feedback and supervision across competencies.

**Table 2:** Developmental stages of psychedelic‑assisted therapy skillfulness.

Stage	Description	Guidance for identifying growth opportunities
Depth/vertical development (Section 6)		
Unskilled	No apparent knowledge of the skill (*Unconsciously incompetent*).	Identify recurring blind spots or patterns and reflect on where new awareness is needed.
Awakening	Becoming aware of the skill (*Consciously incompetent*).	Identify specific gaps and notice where additional learning, practice or support is needed.
Capable	Able to use the skill with effort (*Consciously competent*).	Notice qualities that indicate understanding and skill. Seek feedback to refine the application of the skill.
Integrated	Naturally uses the skill with ease and flow (*Unconsciously competent*).	Notice sustained patterns of integration and consider how to deepen and maintain this capacity.
Breadth/horizontal development (Sections 3)		
Unaware	Knowledge is similar to the general public's; limited familiarity with content.	Identify gaps in understanding and the foundational concepts that require deeper exploration.
Foundational awareness	Basic conceptual understanding with limited ability to apply knowledge in context.	Connect concepts to practical use. Notice where information remains theoretical and identify opportunities for practical application.
Applying knowledge	Can intentionally apply knowledge in dialogue or practice within appropriate professional roles.	Identify where application capacity is inconsistent and strengthen the ability to adapt knowledge to context.
Embodying expertise	Knowledge is integrated and reliably informs decision‐making and collaboration.	Seek opportunities to extend learning through teaching, mentoring or advanced application. Explore complex cases to deepen expertise.
Reflection and Integration (applies to all sections): Seek peer feedback to identify blind spots and consider: What change in my usual routine will help me integrate/embody this skill? How will I address any barriers if they emerge? How will I plan to integrate this new skill? What resources can I reach for when I'm struggling? Growth edges: Areas where a skill is emerging or where there is meaningful room for development. These edges often involve stepping into a new way of being—practices or capacities that may feel unfamiliar, uncertain or vulnerable as they are learned and embodied. *For example, growth edges may include perfectionism, difficulty offering myself compassion, challenges with healthy boundaries, learning how to feel emotions fully, managing expectations, developing capacity for intimacy, expressing emotions outwardly, navigating uncertainty, becoming more assertive, speaking honestly when I do not agree and living more authentically*.		

*Note:* Some competencies are more focused on depth development (e.g., culture and identity); others prioritize breadth‑oriented knowledge integration (e.g., historical, political and regulatory context; evidence‑informed clinical knowledge) and some intentionally integrate both dimensions (e.g., therapeutic presence and trustworthiness).

### 3.3 Competency #1: Therapeutic Presence and Trustworthiness

Therapeutic presence is about showing up with steadiness, compassion and authenticity, in ways clients can feel across emotional, physical, cognitive and spiritual domains. It extends beyond deliberate technique to what is implicitly conveyed through one's way of being—signalling safety, humility and relational attunement.

The programme treats therapeutic presence and trustworthiness as foundational to PaT because these capacities integrate self‑awareness and emotional regulation capacities with applied clinical skill, strengthening alliance and supporting client safety. Therapeutic presence is cultivated through attention to verbal and nonverbal communication [21] and requires both technical competence and reflexive self‑awareness to foster authentic connection [22, 23]. Trustworthiness—expressed through consistency, reliability and transparency—is critical for creating a secure therapeutic environment, particularly given heightened vulnerability in non‑ordinary states of consciousness [24].

### 3.4 Competency #2: Culture and Identity

This competency requires self‑enquiry and self‑management that help practitioners recognize and address biases, assumptions and conditioned patterns shaped by culture and identity (e.g., racial, national, gender, religious and other dimensions). It includes the capacity to give and receive feedback about these dynamics and to navigate differences with openness, humility and readiness to learn. While factual knowledge of diverse histories and practices is important, the primary focus is depth development: exploring one's own positioning, strengthening tolerance for complexity and cultivating awareness of implicit bias in ways that support safety, equity and mutual respect.

Research highlights the importance of cultural and gender inclusivity in PaT for ethical and clinical effectiveness [25, 26]. Intersectionality shapes therapeutic processes and outcomes [27], and culturally responsive practice supports engagement and outcomes [28]. Research on client preferences in psychotherapy further suggests that perceptions of cultural safety and identity attunement—including preferences related to race and gender—are shaped by relational expectations, prior experiences and power dynamics, rather than demographic matching alone [29]. Cultural humility—openness, ongoing self‑reflection and dialogue—is foundational [30]. A harm reduction orientation supports autonomy as policy contexts shift [31, 32], and practitioners must cultivate comfort with ambiguity given the variability of psychedelic experiences [33].

### 3.5 Competency #3: Historical, Political and Regulatory Context

This competency develops practitioners' understanding of the historical, political and regulatory forces that shape PaT practice. Hartogsohn's [34] expanded concept of setting, which includes sociocultural and political dimensions, underscores how these broader contextual factors influence therapeutic processes and outcomes.

Many contemporary psychedelic therapies engage medicines with documented Indigenous lineages [35], while even synthetic substances are often derived from, or informed by, botanical compounds long studied within Indigenous knowledge systems [36]. The War on Drugs and subsequent prohibition have further shaped public perception, access and research trajectories, with lasting consequences for both clients and practitioners. Critiques of the contemporary psychedelic renaissance speak to patterns of colonial extractivism in the commercialization of psychedelic medicines and call for reciprocity through benefit sharing and relational accountability [37].

In parallel, practitioners must navigate rapidly evolving regulatory landscapes, including medicalized treatment models, decriminalization initiatives and alternative regulatory approaches such as those emerging in Oregon and Colorado [38, 39, 40].

### 3.6 Competency #4: Evidence‑Informed Clinical Knowledge

This competency centres on integrating research evidence, clinical expertise, contextual knowledge and consultation to support safe and effective PaT practice. Advances in understanding pharmacological mechanisms [2, 41] support informed decision‑making about dosing, contraindications and risk mitigation [42, 43]. Integrating psychotherapeutic approaches with psychopharmacological knowledge can strengthen outcomes [44, 45]. Therapists who engage critically with emerging research are better equipped to tailor interventions and respond to complexity [46]. Familiarity with complementary modalities (e.g., somatic, transpersonal, arts‑based and other integrative approaches) can expand flexibility and responsiveness when used within scope [47].

### 3.7 Competency #5: Therapeutic Process (Safe, Trauma‑ and Equity‑Informed)

This competency involves co‑creating a safe, predictable, trauma‑ and equity‑informed therapeutic container grounded in unconditional positive regard. It emphasizes shared intentions, clear agreements, consistent boundaries and relational trust across the full PaT arc—screening, preparation, informed consent, dosing and integration—with ethical responsibility understood as continuous rather than phase‑specific.

In PaT, ethical process management is relational and ongoing. Informed consent extends beyond a single event to support autonomy, transparency and shared understanding as experiences and meanings evolve, while attending to power dynamics, expectations and the social, cultural or historical contexts that may shape participants’ sense of choice and safety [48, 49].

Across phases, screening evaluates medical and psychological contraindications and prior experiences with non‑ordinary states of consciousness to support informed decision‑making about suitability and risk [3, 50]. Preparation and dosing integrate biomedical, psychological and contextual considerations, supporting realistic expectations, relational trust and ongoing responsiveness throughout the therapeutic encounter [51, 52, 53].

Integration supports meaning‑making and embodiment across emotional, relational and practical domains [54, 55]. Skills such as somatic attunement, boundary management and awareness of transference and countertransference help sustain safety, accountability and ethical presence as experiences are translated into lived change [6, 56, 57].

## 4 Code of Ethics and Governance Rationale (Summarizing Appendix S3)

This section complements the programme's governance‑oriented Code of Ethics for PaT by outlining the ethical rationale and risk landscape it is designed to address. We situate it within broader ethics literature relevant to PaT—where non‑ordinary states of consciousness can heighten vulnerability, suggestibility and power asymmetries—and describe the domains the Code operationalizes for practice‑based accountability.

The programme's Code of Ethics functions as a governance tool for navigating ethical uncertainty in PaT, where non‑ordinary states of consciousness intensify vulnerability, power asymmetries and interpretive ambiguity—conditions that amplify the ethical demands placed on practitioners and training programmes [58].

In this context, ethical competence extends beyond adherence to formal standards to include the practical capacities required to interpret, negotiate and enact ethical commitments in complex practice settings [59]. Common ethical dilemmas that emerge in PaT include interpretive ambiguity, tensions between professional codes and evolving legal contexts and pressures introduced by rapid regulatory and commercialization shifts, where institutional, financial or legal considerations may at times overshadow ethical reasoning [60].

Communicative ethics approaches—ethics practiced through structured dialogue—such as consultation groups, peer dialogue and supervision—support ethical competence in complex, value‑laden situations where shared reflection is required [61]. Complementing these practice‑level supports, recent field‑wide consensus work suggests ethical priorities for the scaling field, reinforcing the need for clear guardrails that are both practical and revisable over time [62]. These dialogue‑based supports and consensus‑driven priorities position the Code as a practical, shared framework for everyday ethical decision‐making in PaT.

Centred on a relational and developmental understanding of ethics, the Code foregrounds the communicative‐ethics view that ethical competence is relational and enacted, placing shared accountability, rather than rule‑based compliance, at the centre of practice [60]. It offers a principled scaffold for ethical awareness and discernment amid uncertainty, clarifying expectations in high‐risk domains and encouraging consultation, feedback and shared responsibility. When dilemmas arise without precedent, practitioners are expected to gather relevant information, articulate concerns, seek consultation and involve third parties—including relevant authorities—when appropriate.

### 4.1 Ethics Domains and PaT‑Specific Risk Focus

#### 4.1.1 Safety and Harm Prevention

PaT requires robust safety planning given the unpredictability of psychedelic effects and the amplified ethical duty to prevent physical, psychological, relational and cultural harm [5]. The Code emphasizes comprehensive screening, preparation, crisis and emergency response planning and continuity of care and integration supports, aligning with best‑practice calls for consistent safety standards and adverse‑event monitoring as access to PaT expands [46, 63].

#### 4.1.2 Equity, Cultural Accountability and Reciprocity

Ethical PaT practice requires sustained attention to systemic inequities, power dynamics and cultural accountability, including the avoidance of appropriation and the active support of reciprocal relationships with Indigenous communities who steward many psychedelic medicines [64]. Cultural humility and ongoing bias awareness support safety, belonging and therapeutic trust [26, 30, 65], while equity and access concerns remain central as financial, geographic and structural barriers to care persist [4, 66].

#### 4.1.3 Confidentiality, Privacy and Information Stewardship

Therapeutic trust in PaT depends on rigorous confidentiality and privacy practices, particularly given the depth of disclosure and vulnerability common in non‑ordinary states of consciousness. To honour these principles, the Code emphasizes consent‑based information sharing, clear communication about limits of confidentiality and secure stewardship of records and recordings [67, 68].

#### 4.1.4 Transparency, Informed Consent and Professional Representation

PaT requires ongoing, relational informed consent and transparent communication regarding therapeutic processes, risks, practitioner roles and scope, fees, recordings, observers and conditions of care. Ethical transparency supports participant autonomy, informed choice and shared understanding throughout the therapeutic process [48, 69].

#### 4.1.5 Therapeutic Alliance, Power and Boundaries

PaT can heighten transference, countertransference and authority dynamics. For this reason, the Code prioritizes clear boundaries, careful management of power and the avoidance of harmful or dual relationships. Ethical practice depends on ongoing accountability, relational integrity and supervision to maintain the safety of the therapeutic relationship [70, 71].

#### 4.1.6 Touch, Sexual Boundaries and Altered‑State Consent

Therapeutic touch in PaT requires explicit advance consent, a clearly defined purpose and robust accountability processes [56]. The Code gives particular attention to altered‑state consent dynamics, recognizing the risk of suggestibility and boundary drift during non‑ordinary states of consciousness [57]. Sexual boundaries are unequivocal due to the profound power imbalance and vulnerability inherent in PaT, and sexual countertransference requires active supervision and ethical management [72].

#### 4.1.7 Non‑Ordinary States of Consciousness: Heightened Ethical Duty

Non‑ordinary states of consciousness intensify participant vulnerability, suggestibility and relational sensitivity, thereby increasing ethical responsibility. The Code focuses on safeguards related to consent, boundaries, supervision and practitioner humility when working within these states [73, 74].

#### 4.1.8 Financial Ethics and Access to Care

PaT practice requires financial transparency, avoidance of exploitation and attention to inequitable access to care. Cost transparency functions as an ethical safeguard against ambiguity and exploitation in healthcare settings marked by power asymmetries [75], concerns heightened in PaT by rapid commercialization and persistent access barriers [66]. We recognize that calls for transparency may activate concern amid broader financial insecurity and heightened risks of personal data misuse. Here, transparency is framed not as institutional surveillance, but as a relational, consent‑based practice—clear communication about fees, financial arrangements and interests that directly affect access to treatment and the integrity of the therapeutic relationship [75]. Accordingly, the Code addresses fee practices, gifts and conflicts of interest, and affirms advocacy for accessibility, including sliding‑scale or alternative supports where feasible [66].

#### 4.1.9 Professional Competence and Scope Accountability

Practitioner competence requires appropriate training, ongoing professional development and supervision within one's scope of practice. The Code requires accurate representation of qualifications, collaboration across disciplines and timely referral when client needs exceed a practitioner's competence or scope [76]. Attention to culturally situated forms of trauma further underscores the ethical necessity of referral and interdisciplinary collaboration when practitioner competence is limited [28].

#### 4.1.10 Practitioner Self‑Relation and Ethical Sustainability

Ethical PaT practice requires sustained reflexivity, self‑awareness, humility and self‑care. Attention to the practitioner's relationship with self supports ethical sustainability and mitigates risks related to burnout, bias, impairment and unmanaged countertransference [77, 78].

#### 4.1.11 Environmental Stewardship and Responsible Medicine Relations

The Code highlights ethical responsibility towards ecological systems and cultural lineages involved in psychedelic medicine use. This includes attention to the environmental and cultural impacts of sourcing and commercialization, prioritizing sustainability, reciprocity and respectful medicine relations [7, 64].

## 5 Discussion

### 5.1 Operationalizing the Framework

Because PaT training spans regulated and unregulated contexts, the framework centres on transferable design logic rather than jurisdiction‑specific standards.

**Figure 3:**
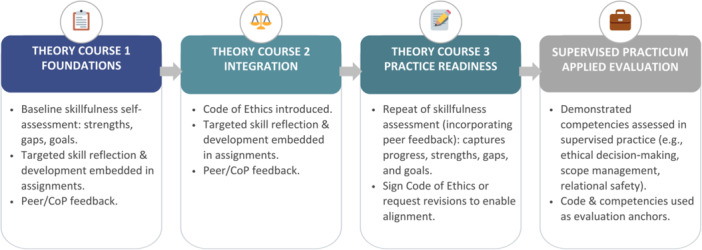
Sequenced integration of skillfulness assessment and ethics. Linear sequencing of the skillfulness assessment (Appendix S2) and Code of Ethics (Appendix S3) across the three theory courses and the practicum. CoP = community of practice, PaT = psychedelic‑assisted therapy.

The following section describes how the competency and ethics framework is enacted across the three theory courses and in supervised practice. Figure 3 shows the linear sequencing of the Skillfulness Assessment (Appendix S2) and the Code of Ethics (Appendix S3) across this pathway. Learners complete the full Skillfulness Assessment at programme entry to establish a developmental baseline, while course‑relevant components are embedded into assignments, rubrics, deliberate‑practice activities and peer/community of practice (CoP) feedback throughout the theory courses. The Code of Ethics is introduced in the second theory course and engaged through dialogue, reflection and supervision. At the end of the final theory course, learners repeat the full Skillfulness Assessment and either sign the Code or propose revisions needed to enable alignment—a readiness checkpoint before practicum.

As students transition into the practice setting, supervisors evaluate the observable application of each competency in real‑world contexts. In practicum, the competencies from the Skillfulness framework, together with the Code of Ethics, function as anchors for assessment, consultation and accountability.

In practice, competence and ethics function as skills enacted in real interactions, not simply as written standards. For this reason, the programme relies on CoP alongside experiential, relational and place‑based learning to foster critical reflection, accountability and safety in PaT practice. This section illustrates how these structures enact the governance‑oriented framework in training; it is not an exhaustive review of pedagogical approaches. CoP serves as the primary container and throughline across theory courses, providing the central relational and pedagogical structure through which the framework is enacted and iteratively refined across cohorts.

### 5.2 CoP

CoPs support competency application by keeping standards *alive* through shared praxis and iterative feedback rather than fixed checklists, aligning with the framework as a *living* scaffold that remains stable for safety while open to refinement [79, 80]. In this context, CoPs serve as structured learning environments where learners engage in facilitated dialogue, case‑based reflection and self‑ and peer‑assessment to examine and practice competencies and ethical commitments.

Within CoPs, learners develop relational readiness and depth‑oriented capacities—reflexivity, emotional regulation, boundary awareness and tolerance for complexity—supported by shared agreements that centre humility, accountability and sustained self‑reflection on identity, power and bias. CoPs make consultation and feedback routine and bring rupture‐and‐repair work into the open, so accountability becomes an ongoing, intentional practice. Skillfulness assessments reinforce this growth, offering feedback on conditioned patterns and providing tools to navigate uncertainty. In these ways, CoPs act as deliberate pedagogical containers that turn communicative ethics into everyday habits of practice, particularly where power, consent and boundaries require continual calibration.

### 5.3 Experiential Learning

Experiential learning involves the development of skills and knowledge through structured reflection on lived experience, including attention to embodied resonance and meaning‑making, and the subsequent application of learning to real‑life situations [81]. In PaT, experiential learning supports practitioners in developing practical understanding, coherence and discernment when accompanying people in non‐ordinary states. When undertaken within clear scope, preparation and supervision, firsthand experience can deepen embodied understanding of altered‐state phenomena, strengthen clinical judgement and support regulated, attuned presence. These capacities are further developed through structured practice, feedback and integration activities [82].

### 5.4 Transformative Learning and Unlearning

Transformative learning supports depth development by surfacing and revising assumptions that shape meaning‑making, power, bias and decision‑making in PaT; often catalysed by *disorienting dilemmas*, it is strengthened by intentional unlearning paired with reflective practice and relearning [19, 83, 84].

### 5.5 Place‑Based and Land‑Based Learning

Place‑based and land‑based learning ground professional formation in relationship to place, ecology and responsibility—an orientation found across many cultural, philosophical and psychospiritual traditions in which land is understood as an active, relational presence rather than a passive backdrop [7, 64]. This orientation is particularly relevant in PaT, where non‑ordinary states can intensify meaning‑making, relational sensitivity and ecological attunement. Evidence linking psychedelic use to increased nature‑relatedness further supports learning‑in‑place as a complementary training structure [85, 86].

In its current form, the programme adopts a place‑based learning approach aligned with cultural safety and humility, while attending carefully to the distinctions between general place‑ or land‑orientation and Indigenous land‑based pedagogies. Indigenous teachings are engaged through relationship‑specific guidance rather than epistemic integration, recognizing that while land‑ and place‑based ways of knowing are found across many traditions, Indigenous land‑based pedagogies articulate distinct ethical, relational and governance obligations that require accountability beyond generalized land‑based orientations [9, 87, 88].

### 5.6 Intersectionality and Inclusivity

Competent PaT practice requires ongoing cultural humility and attention to structural inequities that shape access, safety and trust, particularly in a field whose dominant narratives have historically marginalized Indigenous and racialized contributions [30, 65]. Regular assessment and feedback loops help prevent competency frameworks from becoming rigid or exclusionary by keeping standards responsive to lived experience and equity concerns as psychedelic care moves towards broader implementation [4, 89]. Where competency frameworks are used, ongoing reflexive review is also important because competency systems can function as governance mechanisms that shape practice norms over time [90].

### 5.7 Balancing Structure and Fluidity in CBE

Competency standards function as public‑facing safeguards, but PaT also requires responsiveness to context and personhood. The programme therefore holds a deliberate tension: structure for safety and accountability, alongside flexibility to support relational attunement, creativity and ongoing learning—aligned with longstanding concerns about normalization in competency systems and contemporary calls for context‑sensitive (*adaptive*) standardization in assessment [17, 91]. Programmatic assessment and principle‑based standards offer practical ways to enact this balance while maintaining rigour and transparency [92, 93].

### 5.8 Implementation Across Diverse Contexts

Implementation will require local adaptation. Regulatory requirements, scopes of practice, cultural responsibilities, supervision models and service settings vary considerably across jurisdictions and organizations. For this reason, the framework is intended as a guide for local adaptation rather than a universal competency standard.

Programmes using the framework should review it alongside local regulations, professional standards, cultural guidance and service needs. Commitments related to safety, consent, boundaries, accountability and ethical practice may be broadly relevant, but their application, assessment and governance will vary across settings.

Differences in supervision requirements, documentation practices, informed consent processes, referral pathways and scope of practice are likely to influence implementation. Further evaluation will be needed as the framework is adapted and applied in new settings.

## 6 Conclusion

We describe a practice‑embedded, QI‑derived competency and ethics scaffold developed within a Canadian postgraduate PaT training context. The framework is sufficiently stable to support consistent use, while remaining flexible enough to evolve in response to recurring feedback, emerging evidence and cultural accountability. By integrating breadth (*doing*) and depth (*being*) and pairing competency frameworks with an explicit programme‑level Code of Ethics centred on safety, boundaries, consent, equity and accountability, the model offers a transparent foundation that can be evaluated and adapted across diverse settings.

Although developed over 4 years of iterative feedback, the framework emerged from a single educational and cultural context and has not yet been externally validated. It does not assess clinical outcomes, establish clinical efficacy, imply licensure equivalence or define jurisdiction‑specific training requirements, and should not be assumed to be transferable across settings without contextual adaptation. It offers design principles, ethical guardrails and developmental competencies to support consistency in training and practice while evidence and regulation continue to evolve. Future work should evaluate its feasibility, transferability, implementation conditions and associations with learner, supervisory and clinical outcomes.

## Conflicts of Interest

The authors declare no financial conflicts of interest. Several authors contributed to the programme in roles including faculty, programme leadership, supervision, and/or alumni participation.

## Supporting information


Supporting File 1

Supporting File 2

Supporting File 3

## Data Availability

The framework, standards and Code of Ethics resulting from this QI initiative are available in the supplementary materials.
