# Supplementary material for: Competencies and Ethical Governance for Psychedelic‑Assisted Therapies: A Practice‑Embedded Quality‑Improvement Framework
Source: J Eval Clin Pract. 2026 Aug 10;32(5):e70556. doi: 10.1111/jep.70556 (PMC13456602; doi:10.1111/jep.70556)
Supplement: Supplementary file 1 [file JEP-32-0-s001.docx]

### Appendix 3 Code of Ethics for Psychedelic-assisted Therapy

### Foundations and Development

This Code of Ethics for Psychedelic-Assisted Therapy (PaT) draws on the MAPS Code of Ethics, the Psychedelic Association of Canada Best Practice Criteria, and ongoing consultation with the Roots to Thrive Society for Psychedelic Therapy, an associated service delivery site. Since 2022, it has been shaped through dialogue with students, faculty, alumni, and subject-matter experts within the Vancouver Island University’s Psychedelic-Assisted Therapy Graduate Certificate (PaTGC) program.

The Code carries forward as a living document—one that stays responsive to emerging research, evolving ethical inquiry, regulatory shifts, and the lived experience of practitioners and communities. It is meant to grow through reflection, feedback, and relationship rather than sit fixed on a page.

Ethical practice in PaT isn't a finish line or a static set of rules. It's an ongoing practice of discernment, responsibility, and care. This Code offers shared agreements to help practitioners navigate complex, emotionally charged, and sometimes unpredictable therapeutic terrain. Rather than prescribing rigid answers, it holds a stable ethical container—one that supports clarity, accountability, and integrity while leaving room to meet each situation with presence, humility, and responsiveness.

**Purpose**

A Code of Ethics plays a vital role in shaping and sustaining ethical standards within the emerging field of psychedelic-assisted therapy. This Code supports the credibility and integrity of the PaTGC credentials by:

- offering clear ethical standards to guide professional conduct
- fostering accountability and reflective practice
- safeguarding participants, practitioners, and the public
- supporting the responsible development of the field and those working within it

**Scope and Accountability**

- This Code applies to PaT trainees and credential holders and is intended to be upheld alongside all applicable licensing, regulatory, and legal requirements. Where multiple ethical frameworks apply, practitioners are expected to follow the most protective standard.
- Practitioners commit to integrating evidence-informed practices while honoring the historical, cultural, and spiritual contexts from which psychedelic medicines and practices emerge.

**Ethical Responsibility and Response to Concerns**

- Ethical practice asks for transparency, accountability, and a willingness to address harm when it occurs. Practitioners are expected to engage in ongoing reflection, consultation, and feedback to recognize blind spots and reduce risk.
- When ethical concerns or violations arise and cannot be resolved informally, practitioners are expected to address them with openness and care, including:
- communicating respectfully and directly with those raising concerns, avoiding triangulation unless additional support is needed to ensure safety
- making reasonable efforts to verify relevant facts
- supporting reporting to appropriate regulatory, institutional, or legal authorities when needed to uphold safety, accountability, or compliance

### Commitment

By adopting this Code of Ethics, practitioners affirm their commitment to upholding the principles and standards that follow. Where a practitioner cannot fully agree with a specific provision, they are expected to clearly identify the concern and engage in dialogue aimed at ethical clarity and alignment.

By adopting this Code, practitioners commit to the following ethical principles and standards.

**1. Safety and Harm Prevention**

**Principle:** Practitioners shall prioritize the physical, psychological, relational, and cultural safety of participants before, during, and after PaT.

#### 1.1 Practitioner Responsibilities

- Practitioners shall take reasonable and proactive measures to prevent harm, seek consultation when needed, and respond to foreseeable risks.
- Practitioners shall practice within interprofessional teams when medical monitoring, prescribing, or emergency response falls outside their scope of practice.

#### 1.2 Eligibility and Screening

- Practitioners shall conduct comprehensive medical, psychological, and contextual screening prior to treatment.
- Practitioners shall not initiate treatment when screening indicates that risks outweigh potential benefits or when adequate supports are not in place.
- Practitioners shall consider cultural, relational, and environmental factors, including access to stable housing, social supports, and integration resources.

#### 1.3 Preparation and Informed Care

- Practitioners shall provide thorough preparation, including education about the nature of non‑ordinary states, risks, consent processes, and participant autonomy.
- Participants shall be informed in advance of practitioner availability, backup coverage, and emergency procedures.

#### 1.4 Crisis and Emergency Response

- Practitioners shall maintain a clear crisis response plan and act promptly when participants are at risk of harm. The plan shall include local emergency service contacts, participant emergency contacts, and referral pathways.
- In medical emergencies, practitioners shall contact local emergency services immediately.
- Practitioners shall avoid terminating treatment prior to an agreed-upon endpoint without appropriate planning, communication, and referral or transfer when illness, injury, or safety concerns prevent continuation of the therapeutic relationship.

#### 1.5 Care Between Sessions and Post‑Session Integration

- Practitioners shall clearly communicate the limits of availability between sessions.
- Practitioners shall provide or facilitate access to post‑session integration support appropriate to the intensity of the experience and participant needs.

**2. Equity, Cultural Accountability, and Reciprocity**

**Principle:** Practitioners shall provide equitable, respectful, and culturally responsive care, recognizing the impact of identity, history, power, and systemic inequities on therapeutic relationships. Acknowledging that missteps will occur, practitioners take responsibility for continuous learning, adaptation, and meaningful repair.

### 2.1 Non‑Discrimination

- Practitioners shall not condone or knowingly engage in discrimination. They retain the responsibility to exercise sound clinical judgment to ensure client needs match provider competencies.
- Practitioners shall provide professional services without discrimination on the basis of race, ethnicity, gender, gender identity or expression, sexual orientation, age, religion, disability, national origin, or socioeconomic status.

### 2.2 Self‑Reflection and Unconscious Bias

- Practitioners shall actively examine their own assumptions, biases, and conditioned patterns.
- Practitioners shall engage in ongoing self‑reflection and education to understand how these biases may impact care.
- Practitioners shall practice awareness, openness, and respect in all professional interactions.

### 2.3 Accessibility and Disability Inclusion

- Practitioners shall make reasonable efforts to accommodate physical, psychological, sensory, and cognitive disabilities within the scope of their training and competence. When needs exceed their capacity, practitioners shall seek additional knowledge or provide appropriate referrals.
- Practitioners shall support full inclusion of participants in therapeutic processes to the extent safely and ethically possible.

### 2.4 Respect for Lived Experience

- Practitioners shall honor the uniqueness of each participant’s lived experience, values, belief systems, and paths of healing.
- Practitioners shall remain open to perspectives that differ from their own clinical or personal frameworks.

### 2.5 Power, Privilege, and Positionality

- Practitioners shall remain attentive to power dynamics inherent in therapeutic relationships, particularly where differences in privilege, race, sexuality, gender identity, age, culture, education, ability, or socioeconomic status exist.
- Practitioners shall seek consultation or supervision when power dynamics create uncertainty or risk of harm.

### 2.6 Cultural Humility and Reciprocity

- Practitioners shall approach cultural knowledge with humility, seeking permission and guidance when drawing from traditions outside their own lineage or training.
- Practitioners shall not appropriate cultural teachings or represent them without appropriate context, consent, or relationship.
- Practitioners shall strive to foster reciprocal relationships with cultural teachers and stewards, acknowledging sources and honoring responsibilities that accompany learning.

### 2.7 Curiosity and Integrity

- Practitioners shall be honest about the limits of their understanding.
- Practitioners shall maintain genuine curiosity toward participants’ experiences and perspectives, supporting participants to feel seen, valued, and respected.

## 3. Confidentiality, Privacy, and Information Stewardship

**Principle:** Practitioners shall safeguard participant privacy and confidentiality in accordance with professional, ethical, and legal standards.

### 3.1 Confidentiality Obligations

- Practitioners shall protect the confidentiality of participant information.
- Practitioners shall not disclose identifying information without express consent, except when legally mandated or necessary to prevent serious harm.

### 3.2 Informed Consent and Limits of Confidentiality

- Practitioners shall clearly explain the limits of confidentiality during informed consent.
- Practitioners shall disclose mandated reporting duties or circumstances requiring breach of confidentiality.

### 3.3 Information Sharing

- Practitioners shall obtain written consent before sharing identifying information in consultation or supervision.
- When disclosure is required, practitioners shall share only the minimum information necessary.
- Practitioners shall inform participants of disclosures whenever possible and permissible.

### 3.4 Records and Data Security

- Practitioners shall securely store treatment records and recordings in accordance with the requirements of their professional licensing body and applicable local privacy laws; where unlicensed, practitioners shall follow the privacy laws applicable to the participant’s location.
- Practitioners shall respond promptly to breaches of confidentiality.

### 3.5 Communication Agreements

- Practitioners shall establish clear agreements regarding acceptable communication methods, contact boundaries, and response times.
- Practitioners shall apply these standards across in-person, digital, and remote contexts, including telehealth and electronic communications.

### 3.6 Legal Consultation

- Practitioners shall seek legal counsel when uncertainty exists regarding confidentiality obligations.

## 4. Transparency, Informed Consent, and Professional Representation

**Principle:** Practitioners shall practice with honesty, clarity, transparency, and informed collaboration.

**4.1 Informed Choice and Consent**

- Practitioners shall respect participants’ rights to informed choice.
- Practitioners shall obtain informed consent before initiating new treatments or techniques.
- Practitioners shall honor participants’ right to withdraw consent at any time.

### 4.2 Accurate Representation

- Practitioners shall accurately represent their qualifications, training, and scope of practice.
- Practitioners outside regulated bodies shall be transparent about oversight arrangements.

### 4.3 Financial Transparency

- Practitioners shall disclose fees and payment policies prior to providing services.

### 4.4 Recordings and Observers

- Practitioners shall inform all parties of any recordings and their intended use.
- Practitioners shall obtain explicit written consent for recordings.
- Participants retain the right to refuse non‑essential observers.

### 4.5 Termination and Referrals

- Practitioners shall discuss termination and referral processes during intake.
- Practitioners shall facilitate appropriate referrals when care cannot be ethically or clinically provided.

## 5. Therapeutic Alliance, Power, and Boundaries

**Principle:** Practitioners shall honor the trust placed in them through ethical presence, clear boundaries, and relational responsibility.

### 5.1 Trust and Alliance

- Practitioners shall provide a clear, and where possible, independent process for participants to report discrimination, cultural harm, or ethical violations, while actively mitigating the risk of retaliation or disruption to care.
- Practitioners shall act in a manner that is consistent with clearly defined therapeutic agreements and professional ethical standards, maintaining transparency and accountability in their actions.
- Practitioners shall work with participants to establish clear agreements that support safety, trust, and relational integrity.

### 5.2 Autonomy and Inner Healing Intelligence

- Practitioners shall respect participants’ autonomy and meaning￼making.
- Practitioners shall not impose interpretations or outcomes.

### 5.3 Individualized Care

- Practitioners shall recognize healing as personal and non-linear.
- Practitioners shall prioritize participants’ therapeutic needs over practitioner preferences, except where safety concerns or reasonable clinical judgment require otherwise.

### 5.4 Professional Boundaries

- Practitioners shall maintain appropriate professional boundaries as defined by their licensing or regulatory body; where undefined, practitioners shall refer to recognized professional guidelines to inform their practice.
- Practitioners shall monitor and manage transference and countertransference.

### 5.5 Dual Relationships

- Practitioners shall avoid dual relationships that impair judgment or risk exploitation, while recognizing that community‑based or culturally embedded practices involving dual roles require additional care, transparency, and supervision to ensure relational accountability and ethical integrity.

### 5.6 Research Contexts

- Practitioners shall uphold ethical and scientific integrity when working within research protocols.

## 6. Touch, Sexual Boundaries, and Altered‑State Consent

**Principle:** The approach to touch recognizes that non‑ordinary states of consciousness involve heightened suggestibility and vulnerability, and that consent obtained in these states cannot reliably support the initiation or expansion of physical contact. Therapeutic touch in PaT requires explicit, advance consent established in an ordinary state of consciousness and must never rely on consent obtained in a non‑ordinary state to expand or introduce touch.

### 6.1 Therapeutic Touch

- Practitioners shall obtain explicit, informed consent for therapeutic touch in an ordinary state of consciousness before any medicine session.
- Consent shall include clear agreement regarding:
  - the purpose of touch
  - the specific forms of touch permitted
  - areas of the body that may be contacted
  - boundaries and limits of touch
- Consent shall be documented and reviewed during preparation sessions.

### 6.1.2 Boundaries in Non‑Ordinary States

- Boundaries for therapeutic touch established in an ordinary state of consciousness shall not be expanded, altered, or newly introduced during a non‑ordinary state.
- Practitioners shall not initiate new forms of touch nor exceed previously agreed‑upon boundaries based on consent expressed during a non‑ordinary state of consciousness.

### 6.1.3 Withdrawal of Consent

- Consent for therapeutic touch may be withdrawn at any time, including during a non‑ordinary state of consciousness.
- A withdrawal of consent (explicit or implicit) shall be respected immediately and without question.
- Consent may move only in the direction of “yes to no,” and never from “no to yes,” during a non‑ordinary state.

### 6.1.4 Scope and Accountability

- Therapeutic touch shall occur only within the practitioner’s scope of training and professional competence.
- Practitioners shall terminate touch immediately when it is no longer indicated, requested, or ethically appropriate.
- Practitioners shall maintain transparency and accountability, including the presence of an objective witness (e.g., second practitioner or recording) when feasible.

### 6.2 Sexual Boundaries

- Practitioners shall not engage in sexual or romantic relationships with participants, their partners, or immediate family members during therapy.
- Following termination, practitioners shall not engage in such relationships for a minimum of two years, and longer where required by regulatory guidance.
- Practitioners who enter any relationship with a former client bear full responsibility for demonstrating the absence of exploitation, including that residual trust, vulnerability, or influence from the therapeutic relationship has not been used for the practitioner’s benefit in a way that disadvantages the former client.
- Practitioners shall actively monitor and seek consultation related to sexual countertransference.
- Practitioners shall respect sexual identity and expression without crossing professional boundaries.

## 7. Non‑Ordinary States of Consciousness: Heightened Ethical Duty

**Principle:** Practitioners shall recognize heightened vulnerability and ethical responsibility in non‑ordinary states.

- Practitioners shall prioritize safety, consent, and clear boundaries.
- Practitioners shall monitor intensified transference dynamics.
- Practitioners shall seek supervision to support ethical integrity.
- Practitioners shall approach experiences with humility and openness, without imposing personal belief systems.

## 8. Financial Ethics and Access to Care

**Principle:** Practitioners shall conduct financial practices ethically, transparently, and responsibly.

- Practitioners shall disclose fees and policies clearly.
- Practitioners shall not continue treatment solely for financial gain.
- Practitioners shall exercise discretion when accepting gifts, ensuring they are appropriate, do not present financial or emotional hardship, and do not influence care decisions.
- Practitioners shall strive to increase accessibility where possible.
- Practitioners shall maintain clear and honest business practices.

**9. Professional Competence and Scope Accountability**

**Principle:** Practitioners shall practice within their competence and commit to ongoing development.

- Practitioners shall practice within their scope of training and experience.
- Practitioners shall represent qualifications accurately.
- Practitioners shall provide referrals when care needs exceed their scope of practice or skill competence.
- Practitioners shall engage in continuous learning and accountability, including practicing transparency, avoiding secrecy, and participating in regular consultation within a multidisciplinary professional group.

## 10. Practitioner Self‑Relation and Ethical Sustainability

**Principle:** Ethical practice requires ongoing self‑reflection and care.

- Practitioners shall engage in reflection to maintain a high standard of transparency and integrity.
- Practitioners shall engage in appropriate self‑care practices to support personal awareness, emotional regulation, and professional well‑being.
- Practitioners shall seek support when personal challenges impact care.
- Practitioners shall practice humility in relation to medicine, participants, and cultural traditions.
- Practitioners shall refrain from providing services when personal impairment, illness, or distress compromises their ability to practice safely and ethically and shall seek appropriate support or referral.

## 11. Environmental Stewardship and Responsible Medicine Relations

**Principle:** Practitioners shall act as responsible stewards of medicine, culture, and ecosystems.

- Practitioners shall consider the ecological and cultural impacts of medicine sourcing.
- Practitioners shall prioritize sustainable and ethical sourcing, including synthetic alternatives where appropriate.
- Practitioners shall avoid contributing to exploitative commercialization—using medicines or cultural teachings for profit in ways that cause harm or take advantage.
- Practitioners shall practice, educate, and advocate for conservation, reciprocity, and sustainability.

## Adopting the Code of Ethics

By signaling agreement, practitioners commit to upholding these standards and to identifying any areas requiring clarification or revision to support full ethical alignment.
