# Supplementary material for: Competencies and Ethical Governance for Psychedelic‑Assisted Therapies: A Practice‑Embedded Quality‑Improvement Framework
Source: J Eval Clin Pract. 2026 Aug 10;32(5):e70556. doi: 10.1111/jep.70556 (PMC13456602; doi:10.1111/jep.70556)
Supplement: Supplementary file 2 [file JEP-32-0-s002.docx]

**Appendix 1 Competency and Skillfulness Criteria**

**Purpose**

This Competency Grid articulates the professional capacities, responsibilities, and ways of being that define competent practice in psychedelic‑assisted therapy (PaT). It is intended as a field‑facing framework that clarifies what constitutes responsible, ethical, and skillful practice within an emerging and rapidly evolving domains of care. The grid is not a curriculum outline, instructional manual, or performance checklist. Rather, it names the core domains of competence that practitioners, training programs, regulators, and partner institutions may use as a shared point of reference in shaping education, policy, supervision, and practice standards.

**Developmental Orientation**

Competence in PaT is inherently developmental and non‑linear. Many of the domains described—such as therapeutic presence, ethical attunement, cultural humility, relational discernment, and work in non‑ordinary states of consciousness—unfold over time through reflection, embodied practice, supervision, and lived experience. The presence of a competency within this grid should not be interpreted as implying immediate or complete attainment. Instead, the grid maps the landscape of what mature, integrated practice tends toward over a practitioner’s professional lifespan.

For this reason, the Competency Grid is designed to be read alongside reflective and developmental tools (such as skillfulness or self‑assessment frameworks) without functioning as one itself. It defines what matters in practice; other instruments are better suited to exploring how those capacities are cultivated, embodied, and evaluated over time.

**Ethical and Relational Foundations**

The competencies are grounded in a relational ethic that recognizes psychedelic‑assisted therapy as work conducted in contexts of heightened vulnerability, suggestibility, and power asymmetry. They emphasize safety, consent, boundaries, accountability, cultural humility, and respect for diverse sources of knowledge—including Indigenous, community‑based, embodied, and experiential ways of knowing.

Several competencies explicitly address reciprocity, land‑based awareness, and the historical and political conditions that shape the contemporary PaT landscape. Their inclusion signals that competence in this field extends beyond technical or clinical knowledge to include responsibility for the broader relational, cultural, and ecological contexts in which this work occurs.

**Intended Use**

This Competency Grid may be used to:

- articulate program‑level learning and practice intentions
- inform curriculum design and alignment
- support dialogue with accrediting, regulatory, and partner organizations
- guide supervision, consultation, and professional development
- contribute to shared language within the PaT field about responsible practice

It is not intended to function as a grading rubric, certification checklist, or exhaustive enumeration of all possible skills. Rather, it offers a coherent, values‑informed framework that supports clarity, accountability, and integrity as the field of psychedelic‑assisted therapy continues to mature.

This document is expected to evolve over time in response to emerging evidence, ethical inquiry, regulatory shifts, and dialogue with practitioners, communities, and knowledge holders. Its ongoing refinement is understood as part of the ethical responsibility of stewarding an emerging field of care.

| **CA No.** | **Competency area** | **SC No.** | **Sub-competency** | **LO No.** | **Learning Outcome**  **(skillfulness criteria)**    Competent practitioners in psychedelic‑assisted therapy demonstrate the capacity to: |
| --- | --- | --- | --- | --- | --- |
| CA1 | Therapeutic Presence and Trustworthiness | SC1.1 | Inner Healing Intelligence (IHI) | LO1.1.1 | demonstrate recognition of the client’s inherent capacity to heal and grow within a safe therapeutic relationship. |
|  |  |  |  | LO1.1.2 | support the client’s access to their own IHI through various means such as self-inquiry, somatic tracking and intuition. |
|  |  |  |  | LO1.1.3 | recognize that healing and growth may result from non-linear, unfamiliar, and unexpected experiences. |
|  |  |  |  | LO1.1.4 | describe and relate to the healing power of the natural world, in a reciprocal way, through land-based approaches. |
|  |  | SC1.2 | Emotional Congruence | LO1.2.1 | model emotional congruence and appropriate vulnerability by attuning to a full range of emotional experience while maintaining therapeutic containment and safety. |
|  |  |  |  | LO1.2.2 | demonstrate discernment in drawing on intuitive and other non‑rational ways of knowing, alongside reflective and evidence‑informed practice. |
|  |  |  |  | LO1.2.3 | align verbal expression with genuine values, wants, and needs while maintaining composure and a safe therapeutic environment. |
|  |  |  |  | LO1.2.4 | respond authentically in therapeutic environments through selective, attuned, and clinically appropriate acknowledgement of internal sensations or emotional responses, when such disclosure serves the therapeutic process. |
|  |  | SC1.3 | Groundedness and Self-Regulation | LO1.3.1 | cultivate a calm and consistent therapeutic presence. |
|  |  |  |  | LO1.3.2 | manage their wellbeing and reactions to challenging situations while maintaining work-life balance, self-care, and personal time management. |
|  |  |  |  | LO1.3.3 | proactively structure the therapeutic alliance to ameliorate inconsistency, ambiguity, and insecurity. |
|  |  | SC1.4 | Managing and Leveraging Transference and Countertransference | LO1.4.1 | identify and respond to transference with curiosity and a willingness to explore the client’s experience of self, in the therapeutic relationship. |
|  |  |  |  | LO1.4.2 | identify and manage countertransference to maintain accepted boundaries and ultimately benefit the therapeutic relationship. |
|  |  |  |  | LO1.4.3 | recognize the importance of seeking support from colleagues to address countertransference. |
|  |  |  |  | LO1.4.4 | recognize and effectively address issues associated with erotic transference. |
|  |  |  |  | LO1.4.5 | identify and repair ruptures in the therapeutic relationship. |
|  |  | SC1.5 | Trauma Informed | LO1.5.1 | respond to defensive “safety-seeking” behaviours with curiosity and care, understanding that these behaviours are unprocessed adaptations to adverse life experiences. |
|  |  |  |  | LO1.5.2 | maintain a non-judgemental attitude. |
|  |  |  |  | LO1.5.3 | sustain impeccable interpersonal boundaries. |
|  |  |  |  | LO1.5.4 | welcome the full range of emotional expression. |
|  |  |  |  | LO1.5.5 | reflect others’ strengths and intrinsic value. |
|  |  |  |  | LO1.5.6 | ensure client choice and consent. |
|  |  |  |  | LO1.5.7 | prioritize relational connectedness. |
|  |  |  |  | LO1.5.8 | attend to and validate client concerns and experiences. |
|  |  |  |  | LO1.5.9 | invite clients to engage with, and articulate, the somatic and emotional dimensions of their experience. |
|  |  |  |  | LO1.5.10 | draw on complementary techniques, within scope of practice, to address trauma symptoms. |
|  |  | SC1.6 | Boundaries: Cultivating, Setting, and Upholding | LO1.6.1 | maintain equanimity and compassion when others, particularly clients, are reactive. |
|  |  |  |  | LO1.6.2 | remain differentiated while supporting clients in processing their own experience. |
|  |  |  |  | LO1.6.3 | use techniques to avoid absorbing others’ emotions. |
|  |  |  |  | LO1.6.4 | manage reactivity, such as processing emotional dysregulation at appropriate times and in appropriate ways. |
|  |  |  |  | LO1.6.5 | anticipate and manage situations that may result in boundary rupture. |
|  |  |  |  | LO1.6.6 | communicate boundaries while maintaining unconditional positive regard. |
|  |  |  |  | LO1.6.7 | recognize scope of practice and request assistance when issues outside that scope are encountered. |
|  |  | SC1.7 | Spiritual Intelligence | LO1.7.1 | appreciate the mystery associated with transpersonal experiences. |
|  |  |  |  | LO1.7.2 | explore spiritual themes such as meaning, suffering, intention, and transcendence as well as engaging others in exploring these topics. |
|  |  |  |  | LO1.7.3 | support client’s’ expressions of spiritual experiences without imposing their perspectives on clients. |
|  |  | SC1.8 | Ethics | LO1.8.1 | articulate key ethical challenges in the PaT domain and recognize how these challenges manifest in their own practice. |
|  |  |  |  | LO1.8.2 | manage ethical challenges when they arise (e.g., relational ruptures, boundary violations, and sexual transference). |
|  |  |  |  | LO1.8.3 | identify colleagues who can provide objective perspectives in understanding and assessing ethical challenges. |
|  |  | SC1.9 | Reliability (trustworthiness) | LO1.9.1 | establish relational trust through shared intentions and agreements and holding integrity with them. |
|  |  |  |  | LO1.9.2 | understand and continually cultivate the conditions required to establish a healing relationship and safe container. |
|  |  |  |  | LO1.9.3 | maintain consistency of behaviour such as being reliably punctual, fulfilling commitments, following group agreements, and revising relational agreements when ambiguity or conflict arises. |
| 2 | Culture and Identity | SC2.1 | Humility, Curiosity, and Inclusion | LO2.1.1 | embody genuine interest, openness, and curiosity towards all facets of cultural identity. |
|  |  |  |  | LO2.1.2 | actively seek knowledge and growth from both expected and unexpected sources. |
|  |  |  |  | LO2.1.3 | emphasize the role of the therapist as learner, in client conversations, to better adapt the therapy to client needs. |
|  |  |  |  | LO2.1.4 | cultivate reflexivity towards their implicit biases |
|  |  |  |  | LO2.1.5 | demonstrate humility by explicitly avoiding assumptions about the client’s experiences. |
|  |  |  |  | LO2.1.6 | embody an inclusive and respectful orientation in the PaT setting, including Indigenous and counter-cultural traditions of psychedelic use. |
|  |  | SC2.2 | Providing and Responding to Feedback | LO2.2.1 | receive feedback concerning their biases, assumptions, and ingrained behaviours. |
|  |  |  |  | LO2.2.2 | recognize that while some of their conditioned beliefs and actions may be irritating to others, these external reactions do not diminish their inherent value or self-worth. |
|  |  |  |  | LO2.2.3 | regularly provide appreciative and developmental feedback that offers clear behavioural observations and highlights the impacts of the behaviour. |
|  |  | SC2.3 | Harm Reduction as an Outcome of Health Promotion | LO2.3.1 | proactively self-educate and regularly assess the legal, physical, and psychological risks of psychedelic use. |
|  |  |  |  | LO2.3.2 | regularly provide information about risk mitigation/harm reduction to clients. |
|  |  |  |  | LO2.3.3 | actively cultivate awareness of own stigmas and biases |
|  |  |  |  | LO2.3.4 | recognize and respect that abstinence is one of multiple relationships that a person can have with psychoactive substances. |
|  |  |  |  | LO2.3.5 | recognize that to eradicate stigma, their perspective, language, and actions must transcend and include a harm reduction approach to a wellness promotion approach. |
|  |  | SC2.4 | Intersectionality | LO2.4.1 | develop an awareness of how overlapping identities (e.g., racial, cultural, gender) inform our relationships with one another, social structures, racism, and oppression. |
|  |  |  |  | LO2.4.2 | seek feedback to understand how intersectionality influences the therapeutic alliance so they can mitigate relational safety risks. |
|  |  |  |  | LO2.4.3 | honour others’ intersectionality, understanding that everyone is a unique and complex tapestry woven from multiple identities. |
|  |  |  |  | LO2.4.4 | actively refrain from classifying individuals into a single identity category. |
|  |  |  |  | LO2.4.5 | reflect on assumptions, inquire about experiences and preference, and remain open to learning from clients as experts in their own unique weave of cultural preferences and ways of being. |
|  |  | SC2.5 | Working with Racial and Gender Diversity | LO2.5.1 | develop understanding of diversity as well as racialized and gender-based trauma. |
|  |  |  |  | LO2.5.2 | recognize that fostering a secure therapeutic alliance, where unspoken dynamics are openly addressed particularly regarding cultural tensions, has the potential to facilitate a transformative and healing experience for everyone involved. |
|  |  |  |  | LO2.5.3 | acknowledge that cultural biases may interfere with their ability to effectively communicate. |
|  |  |  |  | LO2.5.4 | seek feedback regarding the therapeutic preferences of their clients, which may include changing therapists. |
|  |  |  |  | LO2.5.5 | identify and address biases and potential discrimination impacting those identifying as 2SLGBTQIA+ via a trauma informed/strengths-based approach. |
|  |  | SC2.6 | Working in Complexity | LO2.6.1 | cultivate a level of comfort with the ambiguity and paradox that may arise in non-ordinary states of consciousness. |
|  |  |  |  | LO2.6.2 | embrace the spontaneous unfolding of the therapeutic process, trusting that challenges may serve as thresholds for fostering trust and facilitating transformation. |
|  |  |  |  | LO2.6.3 | readily shift from established plans/processes to address emerging needs. |
| 3 | Historical, Political, and Regulatory Context | SC3.1 | Political Context of PaT | LO3.1.1 | discuss the traditional use of psychedelics by Indigenous Peoples throughout the world. |
|  |  |  |  | LO3.1.2 | discuss how psychedelics were introduced to the West, the subsequent war on drugs, and how these things inform the contemporary political context. |
|  |  |  |  | LO3.1.3 | describe the socio-political influences that shape the global legal status of synthetic psychedelics and psychedelic plant medicines. |
|  |  | SC3.2 | Historical Context of PaT | LO3.2.1 | evaluate the history of natural and synthetic psychedelics and how this applies to their medical applications. |
|  |  | SC3.3 | Regulatory Knowledge | LO3.3.1 | describe the regulatory landscape for each of the common psychedelic medicines (e.g., psilocybin, MDMA, ketamine, LSD). |
|  |  |  |  | LO3.3.2 | explain the difference between legalization and decriminalization in relation to professional practice impacts. |
|  |  | SC3.4 | Reciprocity | LO3.4.1 | articulate the effects of commercialization of Indigenous medicines on biocultural sustainability, justice, equity, and diversity. |
|  |  |  |  | LO3.4.2 | discuss the importance of benefit sharing, solidarity, and reciprocity with Indigenous Peoples who have stewarded these medicines. |
|  |  |  |  | LO3.4.3 | evaluate ways to engage in respectful acknowledgement of the sources of medicines and counter the negative impacts of commercialization. |
| 4 | Evidence‑  Informed Clinical Knowledge | SC4.1 | Research-informed Knowledge | LO4.1.1 | articulate the different applications for empirical versus theoretical research. |
|  |  |  |  | LO4.1.2 | evaluate how research on PaT, including pharmacology and neuroscience, applies to the use of psychedelics in the treatment of mental health issues. |
|  |  | SC4.2 | Clinically Specific Knowledge | LO4.2.1 | cultivate a working knowledge of the common psychedelics including their neurochemical and biological effects, risk assessment/harm reduction, and therapeutic application. |
|  |  | SC4.3 | Pharmaceutical Understanding | LO4.3.1 | describe the psychological effects, side effects, and reported subjective experience, benefits, and mechanisms of action of MDMA, LSD, ketamine, and psilocybin. |
|  |  |  |  | LO4.3.2 | describe the possible adverse effects associated with MDMA, LSD, ketamine, and psilocybin. |
|  |  |  |  | LO4.3.3 | explain the contraindications for MDMA, LSD, ketamine, and psilocybin. |
|  |  |  |  | LO4.3.4 | discuss the current evidence regarding mechanisms of action associated with microdosing versus psycholytic or psychedelic dosing, within their scope of practice. |
|  |  | SC4.4 | Complementary Techniques | LO4.4.1 | discuss and employ holistic care models that extend beyond the biomedical approach. |
|  |  |  |  | LO4.4.2 | recognize the need for either training in energetic healing or relying on support from those specialists who have energetic healing training. |
|  |  |  |  | LO4.4.3 | articulate how naturopathy, functional/lifestyle medicine, art and music therapy, and somatic and energy healing processes interface with PaT. |
|  |  |  |  | LO4.4.4 | describe the core tenets of Internal Family Systems therapy or other frameworks that acknowledge and engage internal “parts.” |
|  |  |  |  | LO4.4.5 | work comfortably with the multiplicity of the psyche. |
| 5 | Therapeutic Process (Safe, Trauma‑ and Equity‑Informed) | SC5.1 | Alignment and Order | LO5.1.1 | recognize the importance of co-created shared intentions and agreements as fundamental safety measures that are crucial for establishing a strong therapeutic alliance. |
|  |  |  |  | LO5.1.2 | regularly revisit shared intentions and agreements whenever ambiguity surfaces or conflicts arise. |
|  |  |  |  | LO5.1.3 | appreciate the value of incorporating predictable structures in navigating what might otherwise feel like an uncertain journey towards healing. |
|  |  | SC5.2 | Protections and Boundaries | LO5.2.1 | integrate boundaries for individual and collective protection, supporting energetic, emotional, physical, and professional self-care. |
|  |  |  |  | LO5.2.2 | regularly address and resolve boundary violations when they occur in their daily lives. |
|  |  |  |  | LO5.2.3 | realize that the avoidance of setting & respecting boundaries and/or of violations can compromise safety and hamper their ability to maintain a trustworthy therapeutic alliance. |
|  |  | SC5.3 | PaT Screening | LO5.3.1 | identify the signs and symptoms of suicide risk |
|  |  |  |  | LO5.3.2 | assess suicide risk and facilitate a safety plan. |
|  |  |  |  | LO5.3.3 | recognize and screen for the contraindications associated with the major psychedelic medicines. |
|  |  |  |  | LO5.3.4 | describe and differentiate clinical presentation patterns that match specific therapeutic modalities (e.g., group vs. individual, different medicines, microdosing vs psycholytic dosing). |
|  |  | SC5.4 | Preparation to Support PaT | LO5.4.1 | articulate the topics of exploration necessary to prepare the client for the psychedelic session, including preparation for integration supports and processes. |
|  |  |  |  | LO5.4.2 | facilitate the development of intentions and realistic expectations for the PaT experience that focus on process rather than outcomes. |
|  |  |  |  | LO5.4.3 | reflect on the client’s underlying intentions or expectations, helping to make the implicit explicit. |
|  |  |  |  | LO5.4.4 | discuss important considerations in the selection and use of music for the session and subsequent integration. |
|  |  |  |  | LO5.4.5 | articulate the risks and benefits of disclosing their own personal experiences with non-ordinary states. |
|  |  |  |  | LO5.4.6 | describe the essential elements of preparation for the time following PaT (e.g., creating a calm and nurturing environment, working with the neuroplastic window, integration suggestions using physical, written, and artistic practices, etc.). |
|  |  | SC5.5 | PaT Informed Consent | LO5.5.1 | express the multi-faceted aspects of securing comprehensive informed consent, prior to the psychedelic session. |
|  |  |  |  | LO5.5.2 | describe the considerations and approach for obtaining ongoing informed consent regarding therapeutic interventions that pertain to the non-ordinary state experience, during the psychedelic session. |
|  |  | SC5.6 | Medicine Prescribing, Administration, and Monitoring within Multidisciplinary Teams | LO5.6.1 | appraise their own role and scope of practice in relation to the prescribing, administration, and monitoring responsibilities held within the therapeutic team. |
|  |  |  |  | LO5.6.2 | participate in, and collaborate to support, therapeutic team processes that ensure appropriate assessment of eligibility for specific psychedelic medicines, within applicable scopes of practice. |
|  |  |  |  | LO5.6.3 | collaborate with appropriately licensed team members to ensure that relevant client medical history is obtained, documented, and considered in treatment planning. |
|  |  |  |  | LO5.6.4 | recognize diagnostic considerations relevant to PaT and collaborate with licensed professionals whose scope of practice includes diagnostic assessment. |
|  |  |  |  | LO5.6.5 | collaborate with licensed prescribers or medical professionals to evaluate current medications and supplements for relevance to PaT. |
|  |  |  |  | LO5.6.6 | support team‑based assessment of contraindications and interactions in collaboration with professionals authorized to make such determinations. |
|  |  |  |  | LO5.6.7 | contribute to comprehensive, ongoing informed consent processes appropriate to their role, and collaborate with team members to ensure clients are informed of risks and benefits associated with PaT. |
|  |  |  |  | LO5.6.8 | work within a multidisciplinary team that includes members whose scope of practice permits the administration of psychedelic medicines. |
|  |  |  |  | LO5.6.9 | support monitoring processes for specific psychedelic medicines in collaboration with team members whose scope of practice includes medical monitoring. |
|  |  |  |  | LO5.6.10 | participate in team‑based preparation for, and response to, common or expected side effects and adverse events, consistent with their role and scope of practice. |
|  |  |  |  | LO5.6.11 | recognize situations requiring consultation with outside expertise and support appropriate referral or escalation within the therapeutic team. |
|  |  |  |  | LO5.6.12 | communicate effectively with colleagues and external specialists using structured tools such as SBAR (situation, background, assessment, recommendation), as appropriate. |
|  |  | SC5.7 | PaT continuity of care | LO5.7.1 | ensure that therapeutic teams include member(s) whose scope of practice includes receiving client referrals from community care providers. |
|  |  |  |  | LO5.7.2 | ensure that therapeutic teams include member(s) whose scope of practice includes transferring client responsibility to community care providers. |
|  |  |  |  | LO5.7.3 | ensure that their therapeutic teams include member(s) whose scope of practice includes providing referrals for ongoing integration support. |
|  |  | SC5.8 | PaT multidisciplinary teams | LO5.8.1 | appreciate the value of diverse perspectives available when working in multidisciplinary teams. |
|  |  |  |  | LO5.8.2 | seek out, and reflect on, feedback from team members about their actions, aiming for mutuality and collaboration. |
|  |  |  |  | LO5.8.3 | provide appreciative feedback that affirms authentic personal characteristics rather than ego-driven displays.  receive the affirmation of appreciative feedback without ego inflation nor deflation into self-doubt / unworthiness. |
|  |  |  |  | LO5.8.4 | offer constructive feedback, focusing on observations rather than judgments. For instance, if someone's actions elicited an emotional response, describe the action you observed and its impact. |
|  |  | SC5.9 | PaT Core Therapy Skills | LO5.9.1 | confidently guide clients to attend to their bodily sensations. |
|  |  |  |  | LO5.9.2 | attend to their own personal somatic experience to attune to the client’s emotions. |
|  |  |  |  | LO5.9.3 | identify the emerging parts of the client’s self. |
|  |  |  |  | LO5.9.4 | validate the disparate needs and motivations of the client’s parts. |
|  |  |  |  | LO5.9.5 | inquire about the function or experience of the client’s parts. |
|  |  |  |  | LO5.9.6 | normalize the client’s emotions |
|  |  |  |  | LO5.9.7 | promote self-compassion by helping the client to regulate their somatic responses. |
|  |  |  |  | LO5.9.8 | promote self-compassion by encouraging curiosity towards strong emotions. |
|  |  |  |  | LO5.9.9 | promote self-compassion by helping the client to dialogue with the embodied emotion. |
|  |  |  |  | LO5.9.10 | acknowledge client concerns regarding boundaries. |
|  |  |  |  | LO5.9.11 | clarify agreements, roles, and boundaries related to the client’s concern. |
|  |  |  |  | LO5.9.12 | identify alternatives to prohibited activities requested by the client, in order to meet the underlying need(s). |
|  |  |  |  | LO5.9.13 | work with reactive clients by using downregulation and de-escalation approaches in order to continue with the therapeutic process. |
|  |  |  |  | LO5.9.14 | describe and conduct group facilitation to support clients in co-creating a safe space for personal exploration characterized by unconditional positive regard. |
|  |  |  |  | LO5.9.15 | articulate and demonstrate a variety of regulation skills that support group coherence and grounding when group-level activation arises. |
|  |  | SC5.10 | Skillfulness in Non-ordinary States of Consciousness | LO5.10.1 | skillfully dialogue with clients about the value of non-ordinary states of consciousness, mystical experiences, and inner healing intelligence. |
|  |  |  |  | LO5.10.2 | draw on lived experiences with non-ordinary states of consciousness to work with, and through, strong emotional experiences. |
|  |  |  |  | LO5.10.3 | describe the typical subjective phases of the psychedelic experience, which differs according to which medicine is being used. |
|  |  |  |  | LO5.10.4 | articulate the importance of balancing active support for the unfolding therapeutic process and taking a non-directive approach |
|  |  |  |  | LO5.10.5 | describe different approaches for dealing with difficult experiences. |
|  |  |  |  | LO5.10.6 | discuss considerations for supporting transpersonal experiences. |
|  |  | SC5.11 | Integration of Insights into Embodied Behaviours | LO5.11.1 | facilitate the client to articulate significant content of their integration experience. |
|  |  |  |  | LO5.11.2 | encourage creative approaches to promote further integration, particularly non-verbal modalities. |
|  |  |  |  | LO5.11.3 | describe the difference between conceptual knowledge and embodied wisdom and their role in the healing process. |
|  |  |  |  | LO5.11.4 | validate the client’s perspective, responding with questions or statements that invite the client to make meaning of their PaT experience. |
|  |  |  |  | LO5.11.5 | reflect to the client and inquire about values, needs, and/or deep principles that are important to them. |
|  |  |  |  | LO5.11.6 | use, and encourage the use of, visual, kinesthetic, written, and auditory activities to assist with the integration of profound experiences and insights. |
|  |  |  |  | LO5.11.7 | discuss the value of intentional practices (e.g., yoga, meditation, exercise, journalling) and their role in the process of integration. |
|  |  |  |  | LO5.11.8 | explore the relationship between the natural world and healing with respect, humility, and reciprocity. |
|  |  |  |  | LO5.11.9 | guide clients through the experience of disappointment, when their expectations are not met - by tending to the underlying emotional experience, thereby transforming the unmet expectation into a growth opportunity. |
|  |  |  |  | LO5.11.10 | acknowledge the client’s challenging experiences and empathically reflect their concerns. |
|  |  |  |  | LO5.11.11 | invite the client to share their subjective experience of a relational rupture. |
|  |  |  |  | LO5.11.12 | explore how a current day relational rupture presents opportunities to heal psycho-social-spiritual wounds from the past. |
|  |  |  |  | LO5.11.13 | prioritize the continuous interplay of relationships, highlighting the significance of clients projecting their emotions or thoughts onto the therapist. |
|  |  |  |  | LO5.11.14 | actively promote in-depth exploration of these complex relational dynamics. |
