# Supplementary material for: Competencies and Ethical Governance for Psychedelic‑Assisted Therapies: A Practice‑Embedded Quality‑Improvement Framework
Source: J Eval Clin Pract. 2026 Aug 10;32(5):e70556. doi: 10.1111/jep.70556 (PMC13456602; doi:10.1111/jep.70556)
Supplement: Supplementary file 3 [file JEP-32-0-s003.docx]

**Appendix 2 Psychedelic-assisted Therapy Skillfulness Assessment**

The following assessment is designed to help you reflect on your growth in the Psychedelic-assisted therapy Graduate Certificate (PaTGC) program. It supports both learning and professional development and can be used by learners and educators.

This is not a grading tool. Instead, it helps you notice your strengths, areas for growth, and how you are developing over time. Because this program brings together people from different professional backgrounds, each person will engage with the content in ways that fit their role and scope of practice. Growth is non‑linear, and it is normal for some areas to feel more developed than others.

### **Two Areas of Development**

This assessment looks at two types of development:

1. **Depth (Sections 1 & 2)**
   This refers to how you show up—your presence, self‑awareness, ethical grounding, and how you relate to others. Depth reflects the integration of your values and knowledge into everyday practice.
2. **Breadth (Sections 3–5)**
   This refers to what you know—the historical, political, clinical, and procedural foundations of psychedelic‑assisted therapy.

These dimensions together support practitioners who are both self‑aware and well‑informed.

### **How to Use This Assessment**

- For each skill or quality, briefly note one strength and one area you want to grow.
- At the end of each section, choose one skill to focus on more deeply.
- Complete one Active Integration Goal for that chosen skill.
- Focus on how you embody the skill—not on perfection or mastery.
- Expect uneven progress; this is normal.

The table that follows defines the developmental lenses used in the assessment. It is meant to guide reflection, not to score your performance.

| **Developmental Stages of Psychedelic‑Assisted Therapy Skillfulness** | | |
| --- | --- | --- |
| **Depth / Vertical Development (Sections 1–2)** | | |
| **Stage** | **Description** | **Guidance for Identifying Growth Opportunities** |
| **Unskilled** | No apparent knowledge of the skill (*Unconsciously Incompetent*). | Identify recurring blind spots or patterns and reflect on where new awareness is needed. |
| **Awakening** | Becoming aware of the skill (*Consciously Incompetent*). | Identify specific gaps and notice where additional learning, practice, or support is needed. |
| **Capable** | Able to use the skill with effort (*Consciously Competent*). | Notice qualities that indicate understanding and skill. Seek feedback to refine application of the skill. |
| **Integrated** | Naturally uses the skill with ease and flow (*Unconsciously Competent*). | Notice sustained patterns of integration and consider how to deepen and maintain this capacity. |
| **Breadth / Horizontal Development (Sections 3–5)** | | |
| **Stage** | **Description** | **Guidance for Identifying Growth Opportunities** |
| **Unaware** | Knowledge is similar to the general public's; limited familiarity with content. | Identify gaps in understanding and the foundational concepts that require deeper exploration. |
| **Foundational Awareness** | Basic conceptual understanding with limited ability to apply knowledge in context. | Connect concepts to practical use. Notice where information remains theoretical and identify opportunities for practical application. |
| **Applying Knowledge** | Can intentionally apply knowledge in dialogue or practice within appropriate professional roles. | Identify where application capacity is inconsistent and strengthen the ability to adapt knowledge to context. |
| **Embodying Expertise** | Knowledge is integrated and reliably informs decision-making and collaboration. | Seek opportunities to extend learning through teaching, mentoring, or advanced application. Explore complex cases to deepen expertise. |
| **Reflection and Integration (applies to all sections) -** Seek peer feedback to identify blind spots:   - *What change in my usual routine will help me integrate/embody this skill?* - *How will I address any barriers if they emerge?* - *How will I plan to integrate this new skill?* - *What resources can I reach for when I'm struggling?*     **Growth Edges:** Areas where a skill is emerging or where there is meaningful room for development. These edges often involve stepping into a new way of being—practices or capacities that may feel unfamiliar, uncertain, or vulnerable as they are learned and embodied. *For example, growth edges may include perfectionism, difficulty offering myself compassion, challenges with healthy boundaries, learning how to feel emotions fully, managing expectations, developing capacity for intimacy, expressing emotions outwardly, navigating uncertainty, becoming more assertive, speaking honestly when I do not agree, and living more authentically.* | | |

| **Section 1: PaT - Therapeutic Presence and Trustworthiness**  *In this section, emphasis is placed on who we are in relationship, rather than what we do*.  Therapeutic presence is the ability to show up as a compassionate, steady, and trustworthy person. It includes personal integrity, emotional honesty, and the capacity to be fully present—physically, mentally, emotionally, spiritually, and culturally—with clients and colleagues.  This presence isn’t only about skills or techniques. It comes from how we live and relate to ourselves and others every day. When we are grounded, humble, self‑aware, and connected, this naturally shows up in our work.  Over time, what begins as intentional effort gradually becomes ease. As we practice these capacities, they move from something we “try to do” toward qualities that feel natural and integrated. This shift from effort to embodiment is what supports a strong, trustworthy presence in PaT settings. | | |
| --- | --- | --- |
| **SKILLFUL QUALITY** | **SELF-REFLECTION ON EACH QUALITY**  *(Reflecting on your strengths and growth opportunities using the qualities in column 1)*    **IDENTIFY ONE STRENGTH AND ONE GROWTH EDGE PER SECTION** | **ONE ACTIVE INTEGRATION GOAL**  *(describing how you are actively engaging with and investing in the growth edge of the quality you selected in Column 2)*  **DESCRIBE WHAT SUCCESS**  **LOOKS LIKE**  *(How would you look or behave differently once the skill is embodied?)* |
| 1. **Inner Healing Intelligence (IHI)**   *Trusting the client’s inner healing intelligence:* I am confident in the client’s or patient’s innate capacity to heal and grow within a safe, relational container. I prioritize supporting access to inner healing intelligence over reliance on any single model, skill, or intervention, encouraging self-inquiry, somatic tracking, and intuitive knowing alongside reflective understanding.  *Acceptance*: I remain comfortable with unfamiliar, unexpected, or challenging material and honor the nonlinear nature of healing and growth.  *Connection to place-based healing*: I explore and relate to land, nature, and the elements—plants, animals, water, earth, fire, and wind—with respect, humility, and a commitment to reciprocity. |  |  |
| 2. **Emotional Congruence**   *Authenticity:* I role model vulnerability through emotional congruence, expressing and attuning to fear, insecurity, anxiety, grief, and the full range of emotional experience as it arises. Model vulnerability through emotional congruence, expressing and attuning to fear, insecurity, anxiety, grief, and the full range of emotional experience as it arises.  ‑model vulnerability through emotional congruence, expressing and attuning to fear, insecurity, anxiety, grief, and the full range of emotional experience as it arises.  *Self-awareness:* I can discern the inner signal or felt sense of my “gut feeling” (or intuitive knowing) from inner and outer noise, including introjected cultural conditioning or the opinions of others. I express and demonstrate trust in this intuitive knowing.  *Self-expression:* What I express to others aligns with my genuine values, wants, and needs, allowing me to communicate with clarity while maintaining grounding and relational safety.  *Compassionate Witnessing:* When it serves to deepen connection and trust within the therapeutic alliance, I may share sensations or emotions arising in me as I respond to a client’s experience (i.e., somatic resonance). I understand that compassionate witnessing is not about self-focus or making the experience about oneself, but about offering attuned presence that validates another’s lived experience. In doing so, it communicates, “I see you, and I am here with you.” Shared acknowledgment of pain can lessen its isolating impact—what may feel intolerable alone can often become more bearable, and even transformative, when held within a supportive relational field. |  |  |
| 3. **Groundedness & Self-Regulation**   Grounded: I reliably establish a steady therapeutic presence rooted in stillness and embodied awareness. From this grounded state of being, I cultivate an equanimous, contemplative, and mindful presence.  Balance: I consistently attend to my own nervous system activation and wellbeing, maintaining balance through selfcare, healthy boundaries, and sustainable personal time management.  Chaos / Order: I proactively create structure within the development of the therapeutic alliance. This structure functions as a relational safety mechanism that helps contain chaos, ambiguity, and insecurity, supporting a sense of stability and trust. |  |  |
| 4. **Managing and Leveraging Transference and Countertransference**   *Transference:* I identify and respond to transference with curiosity and openness rather than defensiveness. I recognize that projected material arises from the client’s relational history and invite exploration of their experience of self in relationship, rather than correcting, educating, or deflecting.  *Countertransference:* I can recognize and work skillfully with countertransference—moments when my own emotions, memories, or meanings are activated in response to a client—without acting from these impulses. Seeking consultation with colleagues or mentors to process countertransference is a regular and valued practice.  *Erotic transference:* I can recognize erotic transference and countertransference and maintain clear and consistent boundaries, communicating limits in a direct, respectful, and compassionate manner. I understand the distinction between spiritual, relational, and romantic love and the importance of not conflating these experiences.  *Rupture repair:* When ruptures occur in the therapeutic relationship, I take responsibility for making the implicit explicit. I express accountability for my role, support the exploration of relational patterns contributing to the rupture, and seek the resources or consultation necessary to restore trust and repair connection. |  |  |
| 5. **Trauma-Informed**   Recognizing: In my daily encounters, I recognize that defensive or safety-seeking behaviors are adaptive responses to past adversity that were not adequately supported or integrated. When these unconscious patterns arise in myself or others, I respond with curiosity, understanding, and care rather than judgment or criticism.  Safety: I actively cultivate safety and trustworthiness through a nonjudgmental and accepting stance, clear and consistent interpersonal boundaries, openness to the full range of emotional expression, reflection of others’ inherent strengths and goodness, respect for choice and consent, and prioritization of relational connectedness.‑judgmental and accepting stance, clear and consistent interpersonal boundaries, openness to the full range of emotional expression, reflection of others’ inherent strengths and goodness, respect for choice and consent, and prioritization of relational connectedness.  Clinical skills: I acknowledge and validate the client’s stated concerns or lived experience, inviting awareness of bodily sensations and emotions as they arise. When trauma symptoms are present, I engage clients or patients using complementary techniques within my training and scope of practice, supporting regulation, agency, and integration. |  |  |
| 6. **Boundaries: Cultivating, Setting, and Upholding**   *Internal boundaries:* When others are activated, I maintain equanimity and compassion while allowing them to remain responsible for their own experience. I am comfortable allowing emotional processes to unfold without rescuing, absorbing, or over-identifying with others’ emotions. I notice my own reactivity and engage with it consciously and choicefully.  *Relational boundaries:* I anticipate and address situations in which boundaries may be at risk or have been crossed. I communicate boundaries clearly and directly within a context of unconditional positive regard, grounded presence, and compassion. I remain aware of my scope of practice and seek assistance or referral when situations fall outside of it. |  |  |
| 7. **Spiritual Intelligence**   I appreciate the mystery inherent in transpersonal and non‑ordinary states of consciousness. I engage in my own ongoing exploration of meaning, suffering, intention, and transcendence, and I am comfortable supporting others in exploring these dimensions in ways that are authentic to them. I respect diverse expressions of spiritual experience and refrain from imposing my own frameworks, interpretations, or beliefs. |  |  |
| 8. **Ethics**   Understanding: I have a comprehensive understanding of the ethical complexities inherent in PaT and recognize how these challenges may arise in my practice.  Tools: I have access to and make use of ethical decision-making tools and processes when navigating challenges such as relational ruptures, boundary violations, sexual transference, and harm reduction concerns.  Support and supervision: When ethical questions require an objective or external perspective, I reach out to trusted colleagues, supervisors, or mentors for consultation and guidance. |  |  |
| 9. **Reliable (Trustworthiness)**   Creating a healing container: I cultivate relational trust by establishing clear shared intentions and agreements and by attending to the conditions necessary for a safe, supportive, and ethical therapeutic container.  Integrity: I demonstrate integrity by being true to my word, including showing up as agreed, following through on commitments, honoring group agreements, and revisiting or renegotiating agreements when ambiguity or conflict arises. |  |  |

| **Section 2: PaT - Cultivating Proficiency in Culture and Identity**  *This section focuses on self‑awareness, cultural humility, and understanding how we show up in relationship, rather than on being “correct” or achieving mastery.*  Effective PaT therapists are continually developing awareness of their own assumptions, biases, and learned patterns. This includes being able to both offer and receive feedback about *how* their social and cultural identities—such as culture, nationality, ethnicity, race, gender, religion, or other forms of belonging—influence their behavior and impact relationships.  Growing in this area means learning to navigate cultural differences with openness and humility. It calls for more than simply knowing about diversity. It requires staying receptive to learning from lived experiences, unexpected sources, and the people we serve. When we allow these experiences to challenge and expand us, we strengthen our professional, relational, and personal development in PaT practice.  As this capacity continues to deepen, cultural humility becomes more natural and less effortful. Relational awareness integrates into our everyday way of being with others, supporting more authentic, respectful, and attuned therapeutic relationships. | | |
| --- | --- | --- |
| **SKILLFUL QUALITY** | **SELF-REFLECTION ON EACH QUALITY**  *(Reflecting on your strengths and growth opportunities using the qualities in column 1)*    **IDENTIFY ONE STRENGTH AND ONE GROWTH EDGE PER SECTION** | **ONE ACTIVE INTEGRATION GOAL**  *(describing how you are actively engaging with and investing in the growth edge of the quality you selected in Column 2)*  **DESCRIBE WHAT SUCCESS**  **LOOKS LIKE**  *(How would you look or behave differently once the skill is embodied?)* |
| 1. **Humility, Curiosity, and Inclusion**   I strive to maintain an interpersonal stance of genuine interest and openness toward diverse expressions of cultural identity, including ways of being that may be unfamiliar or challenging for me. I remain attentive to moments of discomfort or uncertainty and treat them as opportunities for reflection and learning.  I approach clients with humility, recognizing them as experts in their own lived experience. I remain curious about perspectives that differ from my own and seek to adapt my therapeutic approach in ways that honor each client’s worldview and relational needs. |  |  |
| 2. **Providing and Responding to Feedback**   I am receptive to feedback regarding my biases, assumptions, and conditioned behaviors, recognizing that such feedback offers valuable insight into the impact of my actions. Grounded in a developing sense of self worth, I remain open to feedback even when it evokes discomfort or activation.  I actively seek feedback from others and listen with curiosity to understand its relational impact. When offering feedback, I aim to do so in ways that are appreciative, specific, and grounded in observable behavior, while remaining attentive to values, feelings, and needs. |  |  |
| 3. **Harm Reduction as an outcome of Health Promotion**   *Risk assessment*: I proactively engage in ongoing self‑education (not expecting passive knowledge transfer), and assess the legal, physical, and psychological risks of psychedelic medicines (as well as other substances).    *Knowledge translation:* I readily provide information about risk mitigation/harm reduction to clients/patients (e.g., indications for use, risk versus benefits, safe relationships and settings, drug checking services, safe supply resources, etc.).    *Openness:* I actively examine my own assumptions and potential biases related to substance use, recognizing how stigma can shape clinical judgment. This awareness supports my ability to engage in a broad range of safety‑oriented and wellness‑promoting approaches within PaT practice. |  |  |
| 4. **Intersectionality**   I cultivate awareness of how intersecting social identities—including race, class, gender, sexual orientation, age, ability, ethnicity, and religion—shape lived experience, relational dynamics, and access to power. I remain attentive to how these intersections may influence the therapeutic relationship, including my own assumptions and blind spots.  I approach each person as a complex and evolving whole, resisting the urge to reduce individuals to singular identity categories. I seek feedback, ask questions with care, and remain open to learning how cultural identities and preferences shape each client’s sense of safety and belonging. |  |  |
| 5. **Working with Diversity**   *Self-development:* I am dedicated to advancing my understanding of diversity and racialized and gender-based trauma. I recognize that fostering a secure therapeutic alliance, where unspoken dynamics are openly addressed, particularly in the presence of cultural tensions, has the potential to facilitate a transformative and healing experience for everyone involved.  *Feedback*: I am aware that my cultural biases may interfere with my ability to effectively communicate. With this awareness, I am comfortable asking for feedback and seeking out the preferences of my client/patient.    *Feedback:* I can demonstrate enough comfort with discomfort (if applicable) to address the historical and current impact of discrimination in those identifying as racialized, 2SLGBTQIA+, and all forms of marginalization and oppression in a trauma-informed/strengths-based manner. |  |  |
| 6. **Working in Complexity: Critical Thinking Capacity**   I am comfortable with ambiguity and paradox that arise in PaT settings.    I embrace the spontaneous unfolding of the therapy process, firmly trusting that what may initially seem challenging could serve as a threshold for fostering trust and facilitating empowering shifts in one's belief systems.    I readily pivot from current plans/processes to meet emerging needs effectively. |  |  |

| **Section 3: PaT - Navigating Historical, Political, and Risk Mitigation Realities in the Regulatory Context**  *This section focuses on informed judgment, historical awareness, and ethical decision‑making, rather than on technical expertise or regulatory compliance alone.*  Sound PaT practice requires an understanding of the historical, political, and regulatory forces that shape today’s therapeutic landscape. These forces influence who has access to care, how psychedelic therapies are viewed, and the responsibilities practitioners carry in ensuring safety and ethical practice.  A foundational part of this work is recognizing the diverse origins of psychedelic knowledge, medicines, and healing traditions. It is equally important to integrate this knowledge with care—avoiding objectification, extraction, or appropriation, and instead prioritizing relationships grounded in reciprocity, respect, and accountability.  Engaging with this domain means maintaining an ongoing relationship with history, source traditions, and the communities from which these practices emerge. It also requires discernment: applying knowledge in ways that are culturally and contextually appropriate. This includes understanding the differences and overlaps between decriminalization, legalization, regulation, and harm reduction, and recognizing how each framework shapes the ethical realities of PaT practice as the field continues to evolve. | | |
| --- | --- | --- |
| **SKILLFUL QUALITY** | **SELF-REFLECTION ON EACH QUALITY**  *(Reflecting on your strengths and growth opportunities using the qualities in column 1)*    **IDENTIFY ONE STRENGTH AND ONE GROWTH EDGE PER SECTION** | **ONE ACTIVE INTEGRATION GOAL**  *(describing how you are actively engaging with and investing in the growth edge of the quality you selected in Column 2)*  **DESCRIBE WHAT SUCCESS**  **LOOKS LIKE**  *(How would you look or behave differently once the skill is embodied?)* |
| 1. **Political Context of PaT**   I can accurately discuss the traditional use of psychedelics by Indigenous communities throughout the world, how psychedelics were introduced to the West, the subsequent War on Drugs, and how it informs the current political context.    *Legality*: I can describe the sociopolitical influences that shape the globalization of the legal status of MDMA, ayahuasca, iboga, psilocybin mushrooms, and other psychedelic plant medicines. |  |  |
| 2. **Historical Context of PaT**   I can evaluate the mainstream history of natural and synthetic psychedelics and how it applies to their medical applications. |  |  |
| 3. **Regulatory Knowledge**   I can articulate the regulatory landscape of psychedelic‑assisted therapy as it applies to commonly used medicines (e.g., psilocybin, MDMA, ketamine, LSD), and I understand the distinctions between decriminalization and legalization and their respective implications for professional scope, responsibility, and practice. |  |  |
| 4. **Reciprocity**   *Impact awareness*: I can articulate the impacts of the commercialization of Indigenous medicines on biocultural sustainability, justice, equity, and diversity. I can discuss the importance of benefit sharing, solidarity, and reciprocity with Indigenous Peoples who steward these sacred medicines.    *Respect*: I can evaluate ways to engage in respectful acknowledgement of the sources of medicines and counter the negative impacts of commercialization. |  |  |

| **Section 4: PaT - Evidence-informed Clinical Knowledge**  *This section highlights informed judgment and the thoughtful integration of knowledge, rather than simply collecting information or aiming for expertise alone.*  In PaT, clinical knowledge comes from many sources. This includes scientific research, clinical experience, consultation with experts, and the lived experiences of clients or patients. This “multi‑eyed seeing” approach values multiple ways of knowing. It brings together Western research methods with Indigenous knowledge systems and other culturally rooted teachings, including guidance from Elders and practice‑specific protocols.  Because PaT is evolving quickly within Western medical and clinical settings, practitioners are expected to stay curious and engaged in ongoing learning. This section emphasizes discernment—actively seeking information, evaluating it carefully, and integrating it with intention. Strong clinical judgment develops through continuous inquiry, reflection, and thoughtful application within the complex and rapidly changing landscape of PaT. | | |
| --- | --- | --- |
| **SKILLFUL QUALITY** | **SELF-REFLECTION ON EACH QUALITY**  *(Reflecting on your strengths and growth opportunities using the qualities in column 1)*    **IDENTIFY ONE STRENGTH AND ONE GROWTH EDGE PER SECTION** | **ONE ACTIVE INTEGRATION GOAL**  *(describing how you are actively engaging with and investing in the growth edge of the quality you selected in Column 2)*  **DESCRIBE WHAT SUCCESS**  **LOOKS LIKE**  *(How would you look or behave differently once the skill is embodied?)* |
| 1. **Research-Informed**   I can articulate different applications for empirical versus non-empirical research.    I can evaluate how research on PaT, including pharmacology and neuroscience, applies to the use of psychedelics in the treatment of mental illness. |  |  |
| 2. **Clinically Specific Knowledge**   Based on my scope of practice, I can articulate a working knowledge of common psychedelics, such as their neurochemical and biological effects, risk assessment/harm reduction, and therapeutic application. |  |  |
| 3. **Pharmaceutical Understanding**   *Psychopharmacology*: I can describe the psychological effects, side effects, and reported subjective experience, benefits, and mechanisms of action of MDMA, LSD, Ketamine, and Psilocybin.    *Adverse effects*: I can describe the possible adverse effects of MDMA, Psilocybin, LSD, and Ketamine.    *Contraindications*: I can articulate the contraindications for MDMA, Ketamine, LSD, and Psilocybin.    *Dosing*: I can discuss the current state of evidence regarding the mechanism of microdosing versus psycholytic or PaT dosing. I feel comfortable discussing dosing within the limits of my scope of practice. |  |  |
| 4. **Complementary Techniques & Somatic Energy Modalities**   *Holism:* I am comfortable working with and can describe a holistic care model that extends beyond the biomedical approach.    *Energy healing*: I am either trained in supporting the energetic component of healing or am aware of the need. I am prepared to ask for support from other practitioners who have this training.    *Complementary modalities*: I can articulate how naturopathy, functional/lifestyle medicine, art therapy, and somatic and energy healing processes interface with PaT.    *Parts work:* I can articulate the core tenets of Internal Family Systems therapy or other frameworks that acknowledge and engage internal “parts,” and I am comfortable working with the multiplicity of the psyche. |  |  |

| **Section 5: PaT – A Safe and Supportive Therapeutic Process**  *This section focuses on relational safety, collaboration, and the integrity of the therapeutic process, rather than on technique or procedural control alone.*  A safe and supportive PaT process is created through collaborative engagement between clients or patients and the therapy team. This work is grounded in trauma‑informed and equity‑informed care, and supported by unconditional positive regard—a stance that recognizes each person’s inherent worth and dignity.  Creating this kind of therapeutic container requires intention. It involves co‑creating shared intentions, establishing clear agreements, and setting well‑defined boundaries within a predictable and transparent process. These elements work together to build relational safety, support emotional and nervous‑system regulation, and create the conditions necessary for meaningful therapeutic work to unfold. | | |
| --- | --- | --- |
| **SKILLFUL QUALITY** | **SELF-REFLECTION ON EACH QUALITY**  *(Reflecting on your strengths and growth opportunities using the qualities in column 1)*    **IDENTIFY ONE STRENGTH AND ONE GROWTH EDGE PER SECTION** | **ONE ACTIVE INTEGRATION GOAL**  *(describing how you are actively engaging with and investing in the growth edge of the quality you selected in Column 2)*  **DESCRIBE WHAT SUCCESS**  **LOOKS LIKE**  *(How would you look or behave differently once the skill is embodied?)* |
| 1. **Alignment and Order**   When embarking on a new professional relationship, I recognize the significance of shared intentions and agreements as fundamental safety measures that are crucial to establishing a strong therapeutic alliance. This involves co-creating shared intentions that clarify the purpose behind our collaboration, and this creates explicit agreements that prioritize relational safety. As a consistent practice, I revisit these agreements whenever ambiguity surfaces or conflicts arise, reinforcing the foundation of our professional connection.    *Order amid chaos:* I recognize the value of incorporating predictable structures, providing a sense of order, especially in navigating what might otherwise feel like an uncertain journey toward healing. |  |  |
| 2. **Protections and Boundaries**   I can integrate boundaries for individual and collective protection, supporting energetic, emotional, physical, and professional self-care.    When boundary violations occur in my daily life, I regularly address/resolve them.    I realize that avoidance of boundaries and protections and/or avoidance of addressing violations can compromise needed safety and hamper my ability to maintain a trustworthy therapeutic alliance. |  |  |
| 3. **PaT Screening**   *Suicidality*: I know the signs and symptoms of suicide risk, can assess that risk, and can facilitate a safety plan.    *Contraindications*: I am aware of the contraindications for the different psychedelic medicines and can effectively screen for them.    *Assessment:* I can describe and appropriately differentiate clinical presentation patterns that match certain therapeutic modalities (i.e., group vs individual, different medicines, psychedelic vs psycholytic dosing, etc.). |  |  |
| 4. **Preparation to Support PaT**   *Preparation*: I can articulate the topics of exploration necessary to orient and prepare the client/patient before administration of psychedelics, including preparation for integration supports and processes. For example, I prepare the client/patient for psychological content, highlight the importance of social support, explore coping strategies, and discuss previous non-ordinary state experiences.    *Intention Setting:* In the process of developing intentions for the PaT experience, I am adept at helping clients create realistic expectations that focus on *process over outcomes*. I am honing the skill of shifting the emphasis from a fixation on predetermined outcomes to a focus on the present moment's actions that create a conducive space for the desired intention to unfold.    I reflect on the client’s underlying intentions or expectations, helping to make the implicit explicit, and I invite the client to elaborate on what lies beneath the surface story.    *Music*: I can discuss important considerations in the selection and use of music for the session and subsequent integration.    *Discernment:* I can articulate the risks and benefits of sharing my personal experiences with non-ordinary states.  *Aftercare*: I can describe the essential elements of preparation for the time following PaT (i.e., creating a calm and nurturing environment, working with the neuro-plastic window, integration suggestions using physical, written, and artistic practices, etc.). |  |  |
| 5. **PaT Informed Consent**   *Pre‑session:* I can clearly articulate the core elements of informed consent in PaT, including capacity to consent, voluntary decision‑making, provision of adequate and understandable information, sufficient opportunity for questions, and the right to revoke consent at any time.  *During sessions:* I recognize informed consent as an ongoing process during non‑ordinary states of consciousness and can obtain consent for therapeutic interventions as they arise—including supportive touch, physical safety measures, or deviations from pre‑session agreements—while prioritizing autonomy, clarity, and relational safety. |  |  |
| 6. **Medicine Prescribing, Administration, and Monitoring**   *Team responsibilities*  I understand my own scope of practice and the complementary roles of team members involved in prescribing, medicine administration, and monitoring. I can clearly describe who is responsible for each aspect of care, when consultation is required, and how responsibilities are appropriately shared to support client safety and ethical practice.    *Prescriber responsibilities:* assessing eligibility, obtaining relevant medical history, establishing diagnosis, reviewing current medications and supplements, assessing contraindications and potential interactions, and ensuring informed consent is obtained with clear communication of risks and benefits. They oversee medicine administration, monitor responses specific to the medicine used, anticipate and respond to common or expected side effects and adverse events, and identify when consultation with other professionals is indicated.  *Medical (non-prescriber) responsibilities:* practicing within a non-prescribing scope, obtaining informed consent as appropriate to their role, supporting medicine administration, monitoring physiological and psychological responses consistent with the medicine used, preparing for common side effects or adverse events, and discerning when consultation with prescribers or other team members is required.    *Therapist responsibilities:* maintaining clarity regarding when consultation with nurses, physicians, psychiatrists, or other members of the care team is necessary to support safety, ethical practice, and continuity of care.    *Communication: w*hen consultation is required, I communicate clearly and efficiently using structured frameworks such as SBAR (Situation, Background, Assessment, Recommendation) to support shared understanding and informed decision-making. |  |  |
| 7. **PaT Continuity of Care**   I am comfortable with the following tasks, or am aware that I must have a team member whose scope of practice includes these tasks:   - receiving patient referrals from their long-term care provider - transferring responsibility to a long-term care provider and/or - providing referrals for ongoing integration support |  |  |
| 8. **PaT Multi-disciplinary Teams**   I value the diverse perspectives available when working in multidisciplinary teams.    *Feedback*:  I seek out and actively process feedback from team members about my actions, aiming for mutuality and collaboration.  When appreciative feedback arrives, I pause, breathe, and allow the gratitude to affirm my essence rather than my ego (including when it activates the negative ego states of unworthiness, imposter syndrome, etc.).  When offering feedback and guidance to those in earlier stages of development, I consider and allow for mistakes (without too much risk to client/patients), ensuring I do not compromise an environment of psychological safety. |  |  |
| 9. **PaT Therapy Skills**   *Somatic Attunement:* I am confident in guiding clients to connect with their bodily sensations, and I compassionately observe their feelings by tuning into my own somatic experiences.    *Working with Internal Conflict:* I can identify the emerging parts of the client’s self. I validate the disparate needs and motivations. I inquire about the function or experience of the parts.    *Navigating Strong Emotions:* I acknowledge and normalize the client’s emotions. I promote self-compassion by:   - Helping the client regulate the body. - Encouraging curiosity towards the strong emotion. - Helping the client dialogue with the embodied emotion.   *Boundaries:* I acknowledge the client’s concern. I clarify agreements, roles, and boundaries related to the client’s concern. If a prohibited activity is requested, I clarify what is prohibited, explain why, and explore alternative strategies to support the underlying need.  *Co-Regulating*: I am skillful at working with an activated nervous system by downregulating and de-escalating to establish the connection required to move forward.  *Group Specific:* I can describe and conduct group facilitation to support patients/clients in co-creating a safe space for personal exploration characterized by unconditional positive regard.    I can articulate and demonstrate a variety of regulation skills that support group coherence and grounding when group-level activations arise. |  |  |
| 10. **Skillfulness in Non-Ordinary States of Consciousness**  - *Discussing:* I am skillful in dialogue with clients/patients about the value of non-ordinary states of consciousness, mystical experiences, and inner healing intelligence. - *Experience:* I have experience in non-ordinary states of consciousness and am adept at working with and through strong emotional experiences. - *Informing:* I can describe the typical subjective phases of the medicine experience. - *Timing*: I can articulate the importance of the balance between supporting and engaging, enabling the unfolding process of the client/patient while simply holding space. - *Challenge*s: I can describe best practices in being present to difficult experiences. - *Transpersonal*: I can discuss approaches and considerations in how to support transpersonal experiences. |  |  |
| 11. **Promotion of Integration of Insights to Embodied Behaviours**   I help participants name what they are integrating, and I encourage creativity and actions to promote further integration to highlight non-verbal modalities.   - *Embodied wisdom:* I can describe the difference between conceptual knowledge and embodied wisdom, discussing their role in healing processes. - *Making Meaning:* I appreciate and validate the client’s perspective, and I respond with a question or statement that invites the client to make meaning of their PaT experience. - *Exploring values:* With clients, I reflect or inquire about the qualities of what they may be longing for or that is deeply important to them. - *Varied modalities*: I use and encourage visual, kinesthetic, written, and auditory activities to make conscious space to integrate profound experiences and insights. - *Space:* I can discuss the value of intentional practices (i.e., yoga, meditation, exercise, journaling) and their role in the process of integration. - *An embodied sense of connection to the natural world:* I continue to explore how my and others' healing relates to the land/nature/elements (plants, animals, water, earth, fire, wind, etc.) with respect, humility, and reciprocity. - *Navigating disappointment:* I am proficient in guiding clients through disappointment and transforming it into growth opportunities. I acknowledge and validate the client’s disappointment, and I tend to the emotion beneath it. - *Transforming Ruptures:* I acknowledge the client’s experience and empathically reflect on their concerns. I invite the client to share their subjective experience related to the rupture. I explore how the current rupture presents an opportunity to address unhealed wounds from the past. This proactive approach stems from the recognition that allowing such ruptures to go unaddressed can inadvertently contribute to relational harm. - *Navigating Transference in Therapeutic Practice:* I prioritize the continuous interplay of relationships, highlighting the significance of clients projecting their emotions or thoughts onto the therapist. These projections offer valuable opportunities to confront and overcome relational hurdles within their daily lives. I actively promote in-depth exploration of these intricate dynamics. |  |  |
| Did you find ways to identify if your assessment of self rings true to those living/working with you? Yes ____ No ____    What are you learning as you consider any differences in their perceptions from your own?      **Bridging to your larger vision.** Please stop. Close your eyes. Drop into an intuitive space. Write about where you hope to be in five years in PaT.            **Upon repeat assessments** *(not applicable for your first time through the assessment):* where are you making progress? What past goal(s) require more attention? How might you adjust your strategy to promote success (greater buy-in with meaning making, tapping into desire, etc.)? | | |
